# Genome-Wide Identification and Analysis of NAC Transcription Factor Family in Two Diploid Wild Relatives of Cultivated Sweet Potato Uncovers Potential NAC Genes Related to Drought Tolerance

**DOI:** 10.3389/fgene.2021.744220

**Published:** 2021-11-24

**Authors:** Haifeng Yan, Guohua Ma, Jaime A. Teixeira da Silva, Lihang Qiu, Juan Xu, Huiwen Zhou, Minzheng Wei, Jun Xiong, Mingzhi Li, Shaohuan Zhou, Jianming Wu, Xiuhua Tang

**Affiliations:** ^1^ Sugarcane Research Institute of Guangxi Academy of Agricultural Sciences, Guangxi Key Laboratory of Sugarcane Genetic Improvement and Key Laboratory of Sugarcane Biotechnology and Genetic Improvement (Guangxi), Ministry of Agriculture, Nanning, China; ^2^ Guangdong Provincial Key Laboratory of Applied Botany, South China Botanical Garden, The Chinese Academy of Sciences, Guangzhou, China; ^3^ Independent Researcher, Kagawa-ken, Japan; ^4^ Biological Technology Research Institute, Guangxi Academy of Agricultural Sciences, Nanning, China; ^5^ Cash Crop Institute of Guangxi Academy of Agricultural Sciences, Nanning, China; ^6^ Biodata Biotechnology Co., Ltd, Hefei, China; ^7^ GuangXi Center for Disease Prevention and Control, Nanning, China

**Keywords:** NAC transcription factor, diploid sweet potato, drought stress, expression, 3D structure

## Abstract

NAC (NAM, ATAF1/2, and CUC2) proteins play a pivotal role in modulating plant development and offer protection against biotic and abiotic stresses. Until now, no systematic knowledge of NAC family genes is available for the food security crop, sweet potato. Here, a comprehensive genome-wide survey of NAC domain-containing proteins identified 130 *ItbNAC* and 144 *ItfNAC* genes with full length sequences in the genomes of two diploid wild relatives of cultivated sweet potato, *Ipomoea triloba* and *Ipomoea trifida*, respectively. These genes were physically mapped onto 15 *I. triloba* and 16 *I. trifida* chromosomes, respectively. Phylogenetic analysis divided all 274 NAC proteins into 20 subgroups together with NAC transcription factors (TFs) from *Arabidopsis*. There were 9 and 15 tandem duplication events in the *I. triloba* and *I. trifida* genomes, respectively, indicating an important role of tandem duplication in sweet potato gene expansion and evolution. Moreover, synteny analysis suggested that most NAC genes in the two diploid sweet potato species had a similar origin and evolutionary process. Gene expression patterns based on RNA-Seq data in different tissues and in response to various hormone, biotic or abiotic treatments revealed their possible involvement in organ development and response to various biotic/abiotic stresses. The expression of 36 NAC TFs, which were upregulated in the five tissues and in response to mannitol treatment, was also determined by real-time quantitative polymerase chain reaction (RT-qPCR) in hexaploid cultivated sweet potato exposed to drought stress. Those results largely corroborated the expression profile of mannitol treatment uncovered by the RNA-Seq data. Some significantly up-regulated genes related to drought stress, such as *ItbNAC110*, *ItbNAC114*, *ItfNAC15*, *ItfNAC28*, and especially *ItfNAC62*, which had a conservative spatial conformation with a closely related paralogous gene, *ANAC019*, may be potential candidate genes for a sweet potato drought tolerance breeding program. This analysis provides comprehensive and systematic information about NAC family genes in two diploid wild relatives of cultivated sweet potato, and will provide a blueprint for their functional characterization and exploitation to improve the tolerance of sweet potato to abiotic stresses.

## Introduction

NAM, ATAF1/2, and CUC2 (NAC) transcription factor (TF) family genes, which serve as molecular switches in the temporal and spatial regulation of the expression of their target genes, are an abundant group of plant-specific proteins. NAC TFs are derived from three superfamilies: No apical meristem (NAM), *Arabidopsis* transcription activation factor (ATAF), and Cup-shaped cotyledon (CUC) (Souer et al., 1996; Puranik et al., 2012). NAC TFs often possess a well conserved N-terminal domain with about 150 amino acids and a more variable C-terminal regulatory region (Puranik et al., 2012). The N-terminal domain (NAC domain), which confers DNA-binding ability to NAC TFs, is further divided into five subdomains (A-E), each with different functions: the A subdomain participants in the formation of a functional dimer, B and E subdomains are in charge of the protein’s functional diversification, while subdomains C and D are mainly responsible for DNA binding (Ernst et al., 2004; Chen et al., 2011; Puranik et al., 2012). In addition, the highly dissimilative C-terminal region of NAC TFs can either activate or repress transcription (Peng et al., 2019; Bian et al., 2020).

Increasing studies have illustrated the involvement of NAC TFs in several plant development programs, such as the formation of apical meristems in embryos and flowers (Souer et al., 1996), root development (Xi et al., 2019), fruit ripening (Gao et al., 2018; Martín-Pizarro et al., 2021), cell division and expansion (Kim et al., 2006; Yu, 2019), fiber development (Sun et al., 2018), and leaf senescence (Li et al., 2018). More recently, NAC TFs have received considerable attention as regulators of various abiotic stresses, such as salt (Li et al., 2021), cold (Hou et al., 2020), heat (Guo et al., 2015), and freezing (Ju et al., 2020). In particular, a growing number of results have confirmed that NAC TFs play a key role in response to drought stress. In *Arabidopsis thaliana*, the NAC domain TF *NAC016* binds directly to the promoter of *ABSCISIC ACID*-*RESPONSIVE ELEMENT BINDING PROTEIN1* (*AREB1*) and negatively regulates plant drought tolerance (Sakuraba et al., 2015), while over-expression of either of three homologous genes, *ANAC019*, *ANAC072* or *ANAC055*, in *A. thaliana* conferred significantly fortified drought resistance (Tran et al., 2004). In rice, previous reports revealed that *SNAC1* (Hu et al., 2006), *OsNAC6* (Nakashima et al., 2007), and *OsNAC5* (Song et al., 2011) were related to drought stress and also participated in salt resistance. Recently, drought tolerance-related NAC genes were also reported in tomato (*Solanum lycopersicum*), such as the NAC factor *JUNGBRUNNEN1* (*SlJUB1*), which positively regulated drought tolerance by controlling the expression of three TFs, *SlDREB1*, *SlDREB2* and *SlDELLA* (Thirumalaikumar et al., 2018).

Sweet potato (*Ipomoea batatas* (L.) Lam), which is one of the most important food and industrial crops that is widely cultivated around the world, was ranked as the seventh most outstanding crop globally and fourth in China (Bovell-Benjamin, 2007). Sweet potato is usually cultivated in marginal lands and drought stress is one of the main restrictive factors seriously inhibiting its growth and yield. To date, NAC TFs have been identified in numerous plant species, such as *A. thaliana* (Ooka et al., 2003; Li et al., 2021), rice (*Oryza sativa*) (Nuruzzaman et al., 2010), tobacco (*Nicotiana tabacum*) (Li et al., 2018), tomato (Jin et al., 2020), broomcorn millet (*Panicum miliaceum*) (Shan et al., 2020), and white pear (*Pyrus bretschneideri*) (Gong et al., 2019). Recently, the roles of *IbNAC7* in salt tolerance (Meng et al., 2020) and *IbNAC1* against wounding stress (Chen et al., 2016a; Chen et al., 2016b) have been documented while 12 novel *IbNAC* genes in response to salt stress have been cloned (Meng et al., 2018). Despite these findings, detailed information about the NAC gene family at the genome-wide level has not been available, until now.

Taking advantage of the highly assembled genome of two diploid wild relatives of cultivated sweet potato that were recently published (Wu et al., 2018), in this study, we comprehensively identified and analyzed the NAC TF family for this security crop. A phylogenetic tree was constructed and gene motifs, structure, *cis*-acting elements (CAEs), chromosomal location and interaction networks were also investigated. Moreover, the gene expression patterns in five tissues and under various biotic or abiotic stresses were surveyed using RNA-Seq data, and the expression patterns of select significantly upregulated genes were further determined under drought stress using real-time fluorescence quantitative PCR (RT-qPCR). In addition, 3-dimensional (3D) structures were built and compared between the select *ItfNAC* genes and their paralogous gene in *A. thaliana*. This work provides in-depth and systematic knowledge of NAC TFs in two diploid wild relatives of cultivated sweet potato, and will benefit future functional characterization of the identified NAC genes and facilitate the efficient molecular breeding of polyploid varieties with higher drought tolerance.

## Materials and Methods

### Identification of NAC Family Genes in Two Diploid Wild Relatives of Cultivated Sweet Potato

The *I. trifida* (*Itf*) and *I triloba* (*Itb*) genomes (Wu et al., 2018) were downloaded from the Sweetpotato Genomics Resource (http://sweetpotato.plantbiology.msu.edu). *NAC* gene sequences were searched and downloaded from the *Arabidopsis* Information Resource (TAIR) website (http://www.arabidopsis.org/index.jsp). A hidden Markov model (HMM) profile of the Pfam NAC domain (PF01849) or the NAM domain (PF02365) was employed to identify the sweet potato NAC genes. All *ItfNAC* and *ItbNAC* genes were identified following Li et al. (2020). In brief, candidate sweet potato NAC genes were obtained by searching *I. trifida* and *I. triloba* protein sequences with an HMM (http://hmmer.janelia.org/). Putative *ItfNAC* and *ItbNAC* genes were further verified in the Pfam database (http://pfam.xfam.org/), screened for NAC and NAM domains, and finally confirmed as NAC proteins in sweet potato after redundant sequences were removed with CD-HIT software (http://cd-hit.org/) and confirmed by BLASTP. The chemical properties (including number of amino acids (aa), pI values and molecular weight (MW)) of the *ItfNAC* and *ItbNAC* genes that were identified were obtained from the ExPasy website (http://web.expasy.org/protparam/). The PSORT program was employed to predict the subcellular localization of identified sweet potato NAC proteins (https://psort.hgc.jp/).

### Phylogenetic Tree, Motif and Gene Structure, and Promoter Analysis

A total of 274 NAC proteins in sweet potato and 105 AtNAC proteins were used to construct an unrooted Neighbor-joining (NJ) phylogenetic tree by MEGA6.0 software with 1,000 bootstrap replicates. The parameters used to construct trees followed Yan et al. (2019).

The online program MEME v 4.11.2 (http://meme.nbcr.net/meme/) was used to analyze conserved motifs for the 130 ItaNAC and 144 ItfNAC protein sequences. TBtools (https://github.com/CJ-Chen/TBtools/releases) was used to draw a schematic diagram of the conserved motifs.

The Gene Structure Display Server (GSDS) 2.0 (http://gsds.cbi.pku.edu.cn/) program was used to ascertain the structure of the 274 *NAC* genes in sweet potato. The 2 kb sequences upstream of the start codon ATG of the 274 identified sweet potato *NAC* gene were submitted to the PlantCARE online web server to analyze and identify the CAEs in the promoter (http://bioinformatics.psb.ugent.be/webtools/plantcare/html/).

### Chromosomal Location and Synteny Analysis of *ItfNAC* and *ItbNAC* Genes

The chromosomal location of *ItaNAC* and *ItfNAC* genes were obtained from the Sweetpotato Genomics Resource (http://sweetpotato.plantbiology.msu.edu). TBtools was used to visualize chromosome localizations and duplicated regions of all 274 *ItfNAC* and *ItbNAC* genes. Orthofinder was employed to identify single copy homologous genes in the two wild sweet potato species, and a synteny graph was drawn with TBtools according to the identified single copy homologous genes (Emms and Kelly, 2019). Furthermore, KaKs_Calculator 2.0 was used to calculate synonymous and non-synonymous rates (Ks/Ka) (Wang et al., 2010). The Ks value was further used to calculate the dates of duplication events (T) using equation T = Ks/2r, where r = 8✕10^−9^ for sweet potato (Durbin et al., 1995).

### Identification and Functional Enrichment Analysis of Potential NAC Target Genes in Two Diploid Wild Relatives of Sweet Potato

The 2-kb DNA sequences upstream of the ATG start codon of 274 gene assembled from the sweet potato genome were used to scan NAC TF binding site elements. The potential *ItaNAC* and *ItfNAC* target genes that were predicted with PlantRegMap were used for further pathway enrichment analysis with the KEGG database (https://www.kegg.jp/kegg/pathway.html) (Tian et al., 2020). The hypergeometric Fisher’s exact test (*p* < 0.01) and Benjamini (FDR <0.05) were performed to detect statistically significantly enriched KEGG pathways. The R package ggplot2 was used to draw the top 20 enriched KEGG pathways.

### Interaction Networks of *ItfNAC* and *ItbNAC* Proteins

Protein interaction networks were generated as a co-expression network based on RNA-seq data downloaded from the Sweetpotato Genomics Resource (http://sweetpotato.plantbiology.msu.edu). Samples with an average value of fragments per kilobase of exon per million mapped reads (FPKM) > 10 were used to calculate co-expressed correlation coefficients (*R*) and *p* values. A correlation network between NAC TFs and all genes in the two wild sweet potato species was constructed in R program using the ‘psych’ package with the Pearson method (*R* > 0.5, *p* ≤ 0.05). Finally, Cytoscape version 3.7.2 (https://cytoscape.org/) was employed to draw the co-expression network.

### Cluster Analysis of Expression Data

For the expression profiles of all putative sweet potato *NAC* genes in different tissues (flowers, flower buds, leaves, roots and stems) and exposed to different stress treatments (abscisic acid (ABA), beta-aminobutyric acid (BABA), 6-benzylaminopurine (6-BA), benzothiadiazole S-methyl ester (BITH), gibberellic acid (GA_3_), indole-3-acetic acid (IAA), cold, heat stress, mannitol and NaCl stress), data that was obtained from the Sweetpotato Genomics Resource was mapped with sweet potato gene nucleotide sequences using TopHat version 2.0.8 (http://ccb.jhu.edu/software/tophat/index.shtml). The level of gene expression was calculated by the FPKM. Expression profiles were calculated from a heat map using log2 (FPKM+1) values and shown as a blue-yellow-red gradient by using the heat map package in R version 3.4.0. The threshold (Cq) values of the 36 putative *ItaNAC* and *ItfNAC* genes at 0, 7 and 10 days after drought treatment were determined using RT-qPCR. Expression profiles were generated by Prism 8.0 software (Graphpad, San Diego, CA, United States).

### Plant Materials and Drought Treatment

The twigs of “Guijingshu 8” (2n = 6x = 90) (G8), a widely cultivated sweet potato cultivar in south China, were inserted into plastic trays (3 twigs/tray) in a climate-controlled greenhouse with a 16-h photoperiod at 28 ± 2°C. After cultivation for 10 days, drought stress was induced by withholding watering. Leaves were collected 0, 7, and 10 days after drought stress from nine plants (the leaves of three plants were pooled as one biological replicate, and three biological replicates were collected for each treatment) (Supplementary Figure S1). Collected material was immediately frozen in liquid nitrogen then placed at -80°C until further use.

### RNA Extraction and RT-qPCR Analysis

Total RNA from sweet potato G8 leaves were isolated with the Eastep^®^ Super Total RNA Extraction kit (Promega, Shanghai, China). The quantity and quality of DNA-free total RNA was assessed according to Yan et al. (2019). Successfully synthesized cDNA samples were diluted 1:10 with nuclease-free water and frozen at −20°C until further use.

RT-qPCR was conducted in 96-well plates in an ABI 7500 Real-time system (ABI, Alameda, CA, United States) using the SoAdvanced™ universal SYBR^®^ Green Supermix detection system (Bio-Rad, Hercules, CA, United States). Each RT-qPCR reaction was performed in a 10 µl volume, based on the methods specified by Yan et al. (2019). The sweet potato housekeeping gene *IbGAPDH* (itf08g18140) was used the internal reference gene. The relative gene expression level was calculated by the 2^−ΔΔCT^ method (Livak and Schmittgen, 2001). All RT-qPCR experiments were carried out using three biological replicates for each sample, and three technique replicates for each biological replicate. The gene-specific primers used for RT-qPCR are listed in Supplementary Table S1.

### 3D Structure Construction and Comparison Between ANAC019 and ItfNAC62

The 3D structures of ANAC019 and ItfNAC62 were constructed by homology modeling, as follows. A homologous protein search was first performed using the MPI Bioinformatics Toolkit (http://toolkit. tuebingen.mpg.de) (Alva et al., 2016) and proteins with the highest homology (>60% homology) were then selected as templates to construct the ANAC019 and ItfNAC62 3D structures by MOE2019 software (Chemical Computing Group, Quebec, Canada). Two online tools SAVES, v6.0 (https://servicesn.mbi.ucla.edu/SAVES) and PROSA (https://prosa.services.came.sbg.ac.at), were used to inspect the reliability of constructed 3D structures. In addition, MOE2019 software was employed to compare structural similarities and differences between ANAC019 and ItfNAC62.

### Molecular Dynamics Simulation of ANAC019 and ItfNAC62

Desmond version 2020 (D. E. Shaw Research, New York, NY, United States) was used and the force field of OPLS3e was employed for molecular dynamics (MD) simulation. The TIP3 water model was adopted to solvate the system. The SHAKE algorithm was employed to constrain the geometric configuration of water molecules, as well as the bond length and bond angle of heavy atoms. The particle grid Ewald method with periodic boundary conditions was used to numerically simulate a long-range electrostatic field. The system was balanced using the NPT system at 300 K (temperature) and 1.0 bar (pressure). The Berendsen coupling algorithm was employed to couple the temperature and pressure parameters. During final system preparation, a 200 ns production run was performed with a time step of 1.2 fs, a track was recorded every 200 ps, and a total of 1,000 frames were recorded. Root-mean-square deviation (RMSD) values of the skeleton atom were calculated.

### Statistical Analysis

Statistical analysis was performed using SPSS 19.0 (IBM Corp, Armonk, NY, United States) to analyze the RT-qPCR data. One-way analysis of variance (ANOVA) was carried out, and significance between treatment means was assessed by Duncan’s multiple range test at *p* < 0.05.

## Results

### Identification and Phylogenetic Analysis of the NAC Gene Family in Two Diploid Wild Relatives of Cultivated Sweet Potato

We retrieved a total of 283 non-redundant NAC domain-containing TFs from the *I. triloba* and *I. trifida* genomes by local BLASTP and HMMER searches, and these 283 sweet potato NAC TFs were renamed as *ItbNAC1* to *ItbNAC132*, and *ItfNAC1* to *ItfNAC151* based on their chromosomal localization in *I. triloba* and *I. trifida* genomes, respectively. Among all of the identified 283 NAC TFs, the 274 genes including 130 *ItbNACs* and 144 *ItfNACs* had a full-length coding sequence and were used for the further analysis. The aa of 130 ItbNACs ranged from 86 to 896 (average = 343.5 aa) and between 90 and 835 (average = 333.37 aa) for 144 ItfNACs. The pI values of 130 ItbNACs ranged from 4.03 to 11.57, and from 4.07 to 10.66 for 144 ItfNACs. The MW of 130 ItbNACs ranged from 9.86 to 101.97 kDa, and from 10.13 to 93.15 kDa for 144 ItfNACs. Most of the identified NAC proteins were localized in the nucleus, i.e., 98 ItbNACs and 109 ItfNACs, followed by 20 ItbNACs and 19 ItfNACs that were localized in the cytoplasm. In addition, 3 ItbNACs and ItfNACs were localized in mitochondria, and 4 ItbNACs and 7 ItfNACs were localized in chloroplasts (Supplementary Tables S2, S3).

**FIGURE 1:**
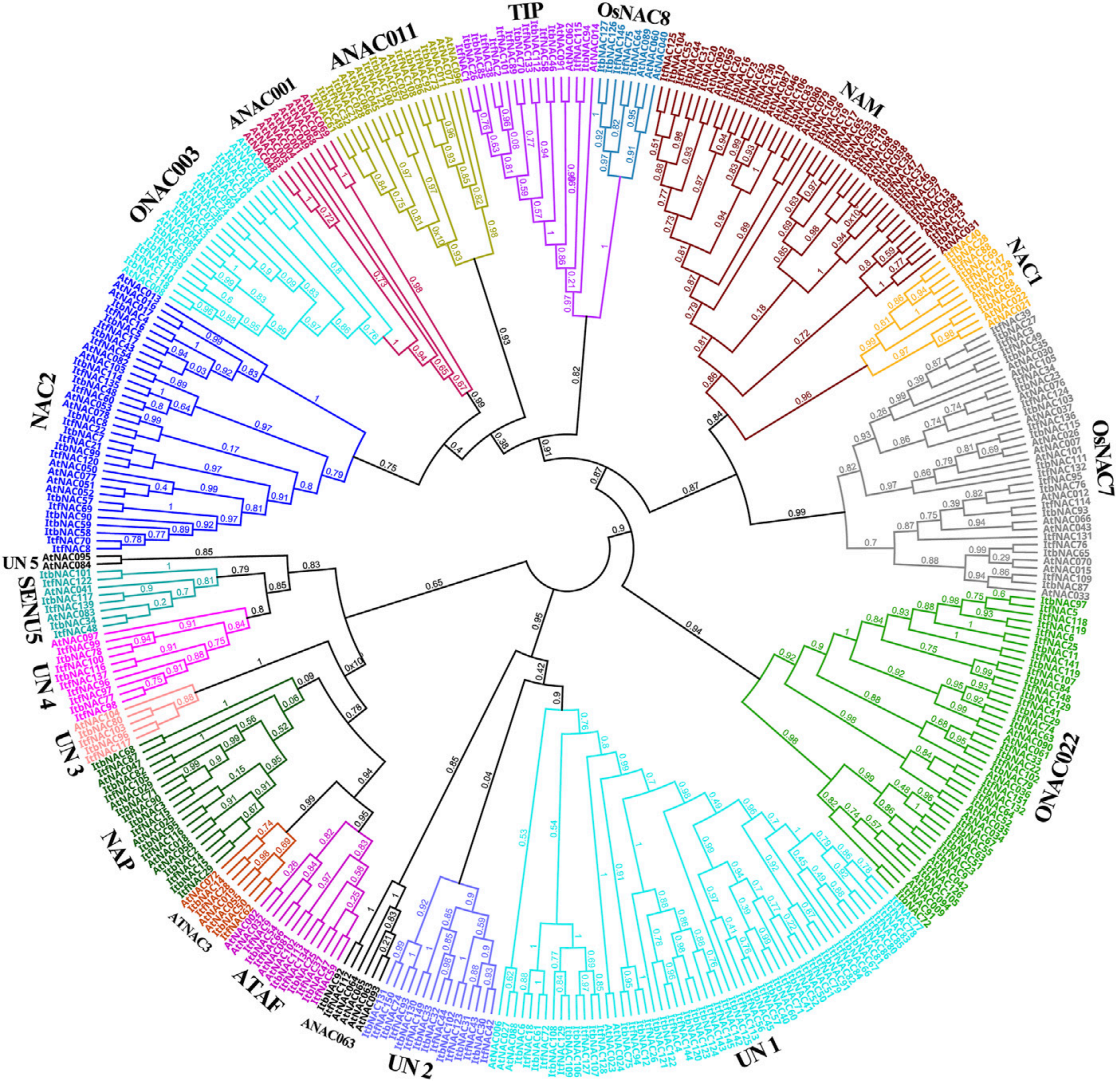
Phylogenetic tree of the NAC family proteins from two diploid wild sweet potato species and Arabidopsis thaliana. 274 NAC proteins from sweet potato and 105 representative AtNAC proteins from *A. thaliana* were used to construct a Neighbor-joining phylogenetic tree by MEGA6.0 software with 1,000 bootstrap replicates. All 274 NAC proteins were classified into 20 subgroups.

An unrooted NJ phylogenetic tree was constructed between 274 sweet potato and 105 *A. thaliana* NAC proteins to understand their evolutionary relationships. As shown in Figure 1, sweet potato NAC TFs were classified into 20 subgroups based on their sequence similarity and topology. Unclassified 1 (UN1) was the largest group with a total of 55 TFs, followed by NAM with 54 TFs, and ONAC022 with 43 TFs. Curiously, UN2 only contained sweet potato NAC TFs, indicating their crucial evolutionary roles in the sweet potato genome.

### Gene Motif and Structure Analysis of *ItfNAC* and *ItbNAC* Genes

In order to explore genetic diversification in sweet potato, the conserved motifs of the 274 NAC genes were investigated. In total, 10 conserved motifs were identified by the MEME program (Supplementary Figure S2B). NAC protein sequences in the same group had a similar conserved motif composition and order. Most of the NAC genes contained four conserved motifs in a similar order. Out of 274 *NAC* genes, 259 shared conserved motif 3. Additionally, conserved motif 9 was unique to group 8, suggesting that its functional divergence contributed to this group.

To further understand the functions of *NAC* genes and their evolution in sweet potato, gene structural diversity was analyzed (Supplementary Figure S2C). Most of the members in the same group shared similar exon/intron structures, including members in groups (G) 1, 2, 6, 7, 8, 9, 10, 11, 13, and 14. However, a few members in individual groups (G3, G4, G5, G12, and G15) had slightly different exon/intron structures. In summary, 28 sweet potato NAC genes had one exon and zero introns while 136 NAC genes had three exons and two introns. *ItfNAC44* in G4 had the largest number of exons/introns, i.e., 11 exons and 10 introns, followed by *ItbNAC32* (in G4) and *ItbNAC51* (in G10), with 10 exons and 9 introns each.

### Promoter Analysis of *ItfNAC* and *ItbNAC* Genes

To investigate the potential transcriptional regulation mechanism of *NAC* genes in sweet potato, 2-kb DNA sequences upstream of the start codon ATG were submitted to the PlantCARE web server to confirm the presence of CAEs. A total of 22 CAEs were found (Supplementary Figure S3). Most of the identified CAEs were associated with abiotic response, including wound response (accounting for 89.05% of all *NAC* genes identified), sulfur response (85.4%), stress response (83.58%), anaerobic induction (80.66%), low temperature response (75.55%), drought response (55.47%), dehydration response (34.67%), sucrose response (18.61%), antioxidant response (2.19%), desiccation response (1.82%), and heat response (0.36%). Other CAEs that were identified were involved in hormone response, including to gibberellin (GA), ABA, methyl jasmonate (MeJA), ethylene, auxin and salicylic acid (SA). GA-responsive elements were found in 122 *ItbNAC* genes and in 129 *ItfNAC* genes and covered the largest proportion (91.6%) of the identified *NAC* genes. ABA-responsive elements, which accounted for 83.94% of the identified *NAC* genes, were found in 108 *ItbNAC* genes and in 122 *ItfNAC* genes. In addition, three tissue-specific CAEs were found in some identified *NAC* genes, such as meristem-, seed-, and endosperm-specific CAEs. Apart from these CAEs, light-responsive elements were also found and were distributed in all of the identified *NAC* genes.

### Chromosomal Location and Synteny Analysis of *ItfNAC* and *ItbNAC* Genes

**FIGURE 2:**
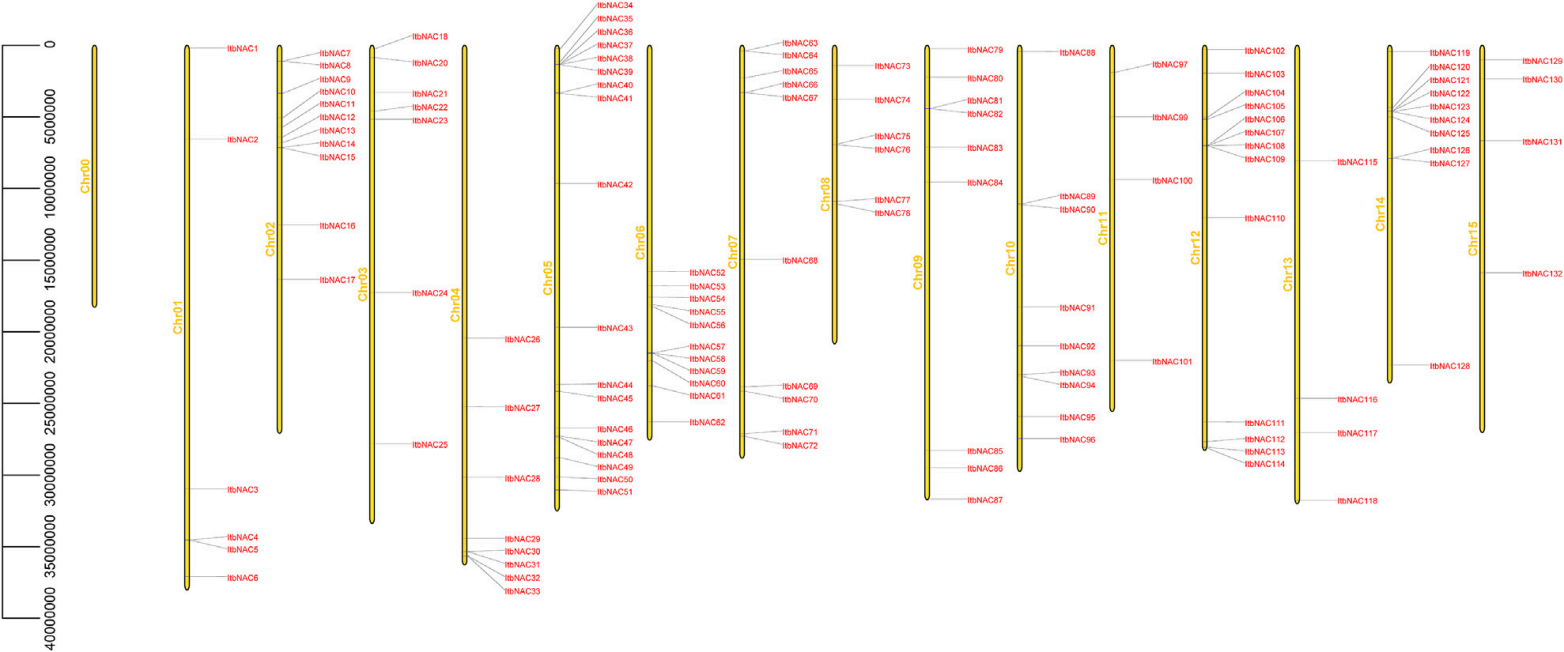
Chromosomal location of ItbNAC genes. Yellow bars on the left side represent chromosomes, and gene ID on the right side represents its location mapped on the chromosome. 130 *ItaNAC* genes were unevenly distributed throughout 15 *Ipomoea triloba* chromosomes.

**FIGURE 3:**
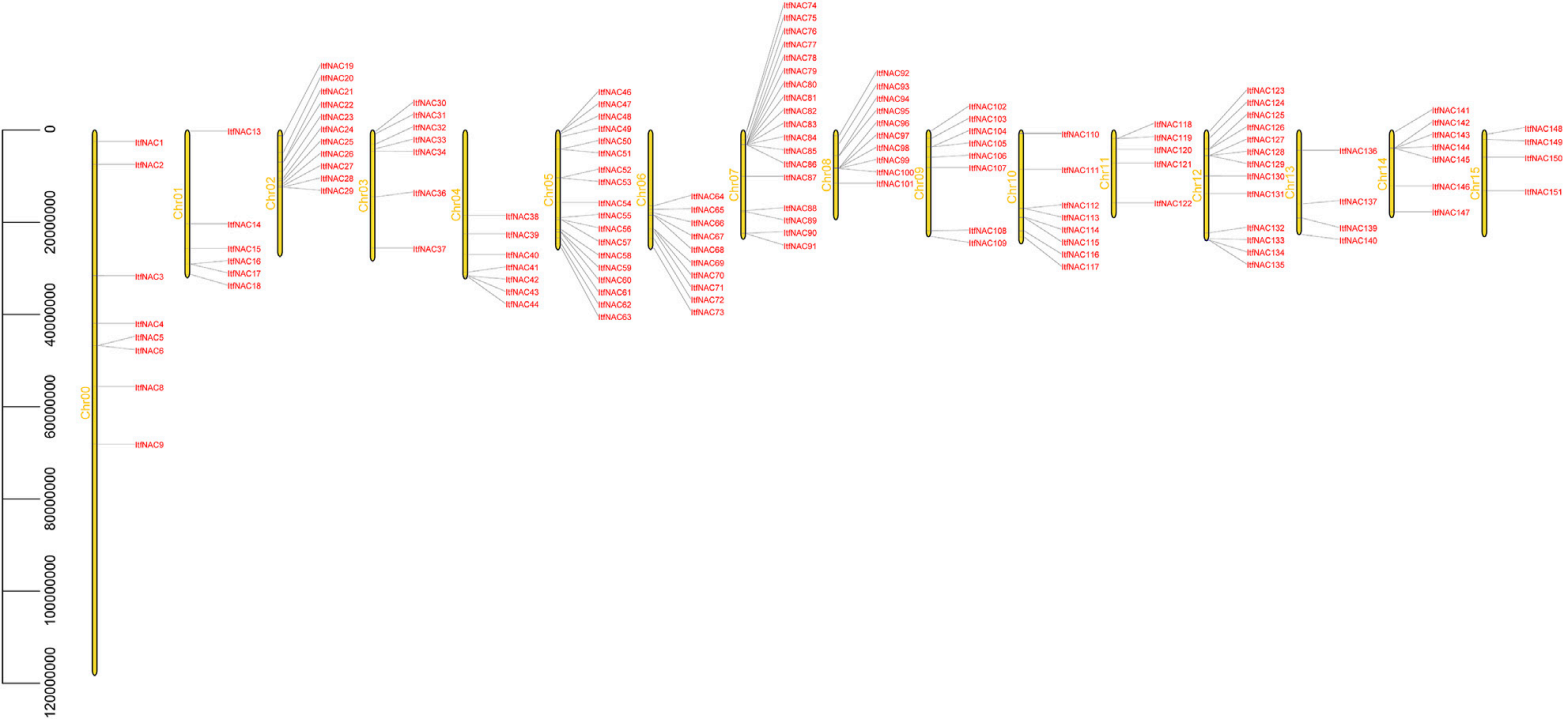
Chromosomal location of ItfNAC genes. Yellow bars on the left side represent chromosomes, and gene ID on the right side represents its location mapped on the chromosome. 144 *ItfNAC* genes were unevenly distributed across 16 *Ipomoea trifida* chromosomes.

To better understand the location of the identified *NAC* genes on diploid sweet potato chromosomes, the 130 and 144 *NAC* genes were mapped on *I. triloba* and *I. trifida* chromosomes, respectively. The 130 *NAC* genes were unevenly distributed throughout 15 *I. triloba* chromosomes, and chromosome 5 contained the majority of *NAC* genes (18 genes, 13.84%), followed by chromosome 12, which contained 13 *NAC* genes (10%), whereas the chromosomes 0 had no gene contained (Figure 2). Similarly, 144 *NAC* genes were unevenly distributed across 16 *I. trifida* chromosomes: chromosomes 5 and 7 contained most *NAC* genes (18 genes each, 12.5%), followed by chromosome 12 with 13 genes (9.03%), while chromosome 13 and 15 contained the fewest *NAC* genes (4 genes each, 2.78%) (Figure 3). In general, gene duplication, including tandem, segmental and whole-genome duplication, are the essential driving forces in the evolution and expansion of gene families. A total of 21 *NAC* genes resulted from 9 tandem duplication events in *I. triloba* and 41 *NAC* genes resulted from 15 tandem duplication events in *I. trifida*. Chromosome 12 in *I. triloba* contains the most *NAC* gene clusters, including *ItbNAC106*, *ItbNAC107*, *ItbNAC108*, and *ItbNAC109* (Figure 2) while chromosome seven in *I. trifida* contains 10 clustered *NAC* genes (Figure 3). Segmental and whole-genome duplication events were not identified.

**FIGURE 4:**
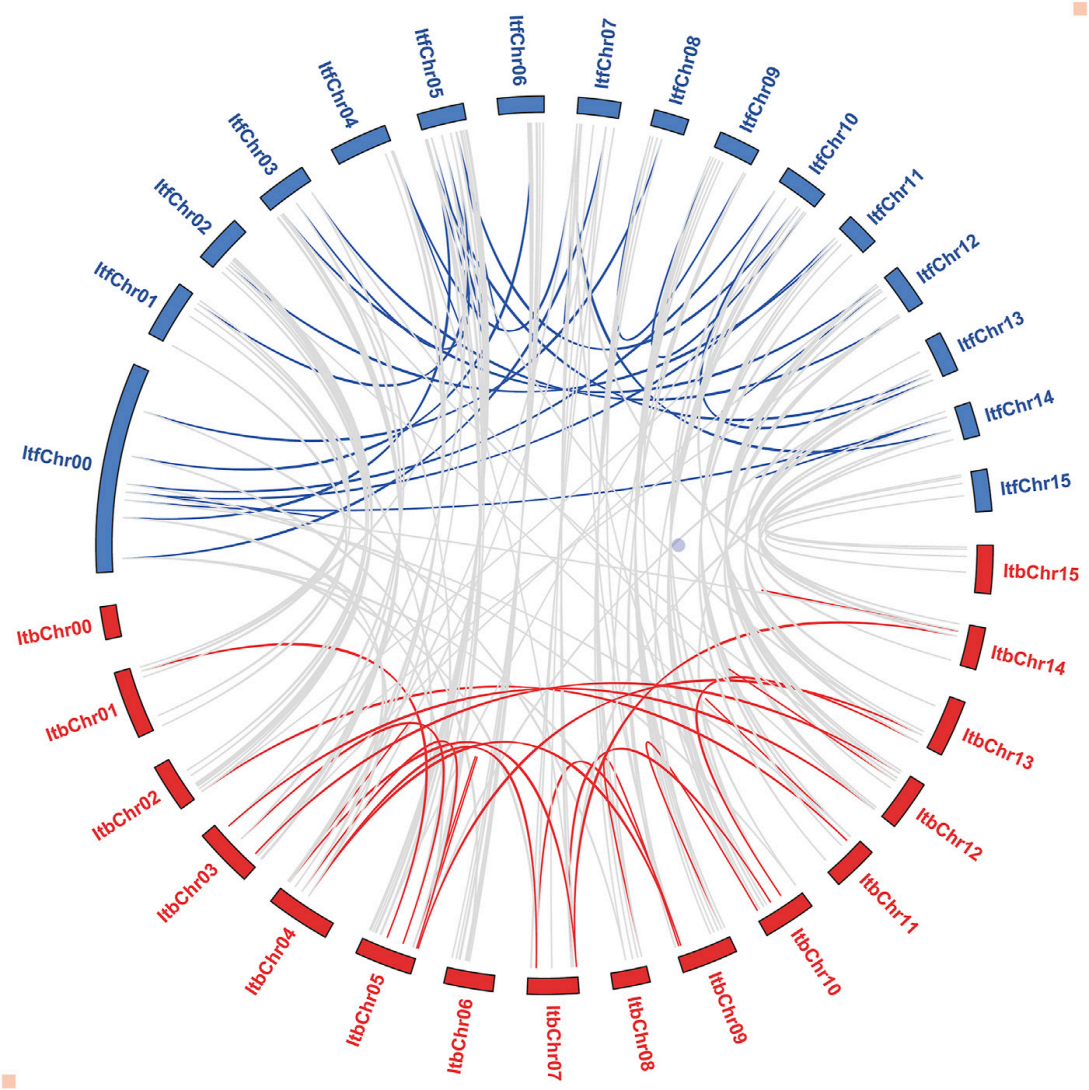
Syntenic relationship among ItbNAC and ItfNAC genes. Red and blue curves represent the synteny relationship of *NAC* genes in the *Ipomoea triloba* and *Ipomoea trifida* genomes, respectively. Grey curves represent the synteny relationship among *ItbNAC* and *ItfNAC* genes.

According to the synteny analysis performed by Orthofinder, there was a high collinearity relationship among most of the *I. triloba* and *I. trifida* chromosomes, suggesting that most of the *NAC* genes in both species had a similar origin and evolutionary process. However, the collinearity relationship for chromosomes 4 and 5 between both species was inconsistent, suggesting that chromosome rearrangement events occurred in these chromosomes (Figure 4). Furthermore, the selection pressure and divergence time of the duplicated events were estimated by Ka (non-synonymous) and Ks (synonymous) ratio. Among the 108 gene pairs, most evolved under purifying selection (Ka/Ks < 1.0), except for one gene pair (*ItbNAC114/ItfNAC135*), which evolved under positive selection (Ka/Ks > 1.0). The doubling time of most genes within each species was between 50 and 90 million years ago (Mya), and the divergence time between both species was traced to about 3.9 Mya (Supplementary Material S1).

### Prediction and Functional Enrichment Analysis of Potential *ItfNAC* and *ItbNAC* Target Genes

As important regulators of gene expression, NAC TFs often regulate the expression of target genes by binding to their promoter’s CAEs. After scanning the 2-kb DNA sequences upstream of the ATG start codon of 274 genes assembled in the *I. triloba* and *I. trifida* genomes, the obtained potential target genes were then analyzed using the KEGG database. As shown in Supplementary Figure S4, the top enriched KEGG pathways of *ItbNAC* target genes included cysteine and methionine metabolism, glycerophospholipid metabolism, carbon fixation in photosynthetic organisms, and SNARE interactions in vesicular transport. The top enriched KEGG pathways of *ItfNAC* target genes included endocytosis, RNA degradation, oxidative phosphorylation, and pentose and glucuronate interconversions (Supplementary Figure S5). These results indicate that *NAC* genes in sweet potato are closely involved in metabolism, processing of genetic information, as well as in other biological pathways.

### Interaction Networks of ItfNAC and ItbNAC Proteins

**FIGURE 5:**
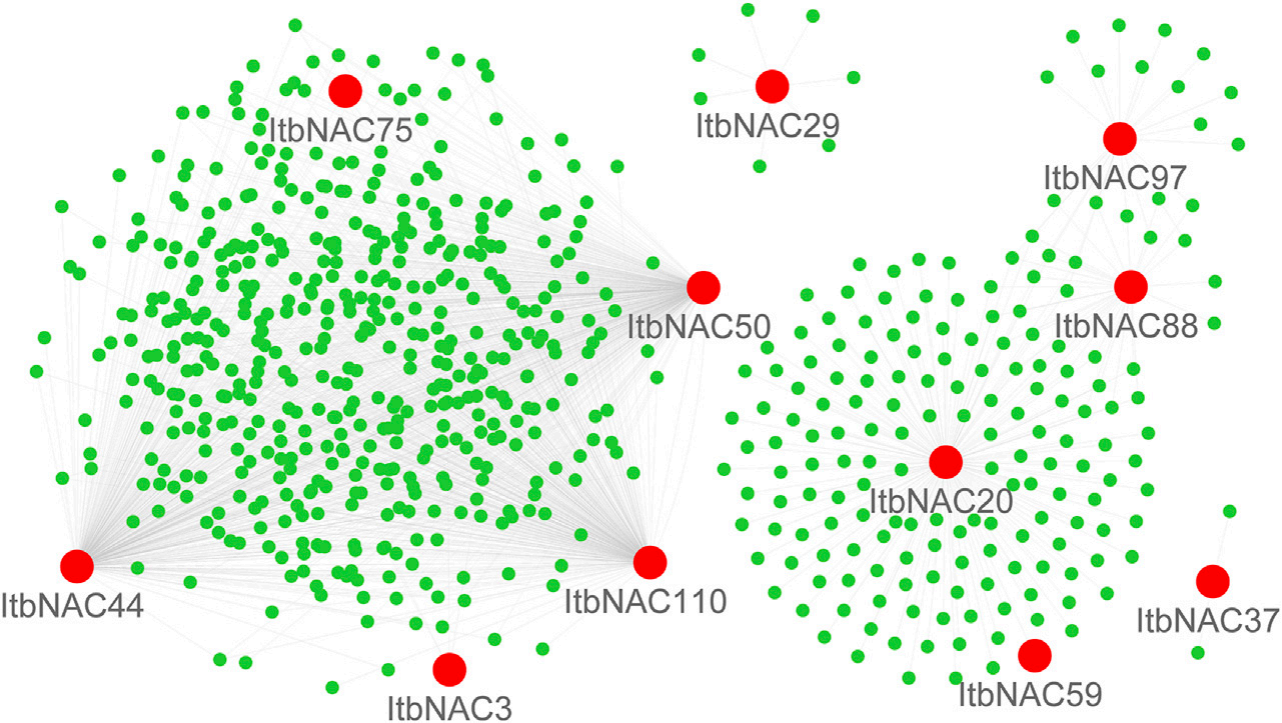
Interaction networks of ItbNAC genes. The red circle indicates NAC family proteins, and green circle indicates other proteins.

**FIGURE 6:**
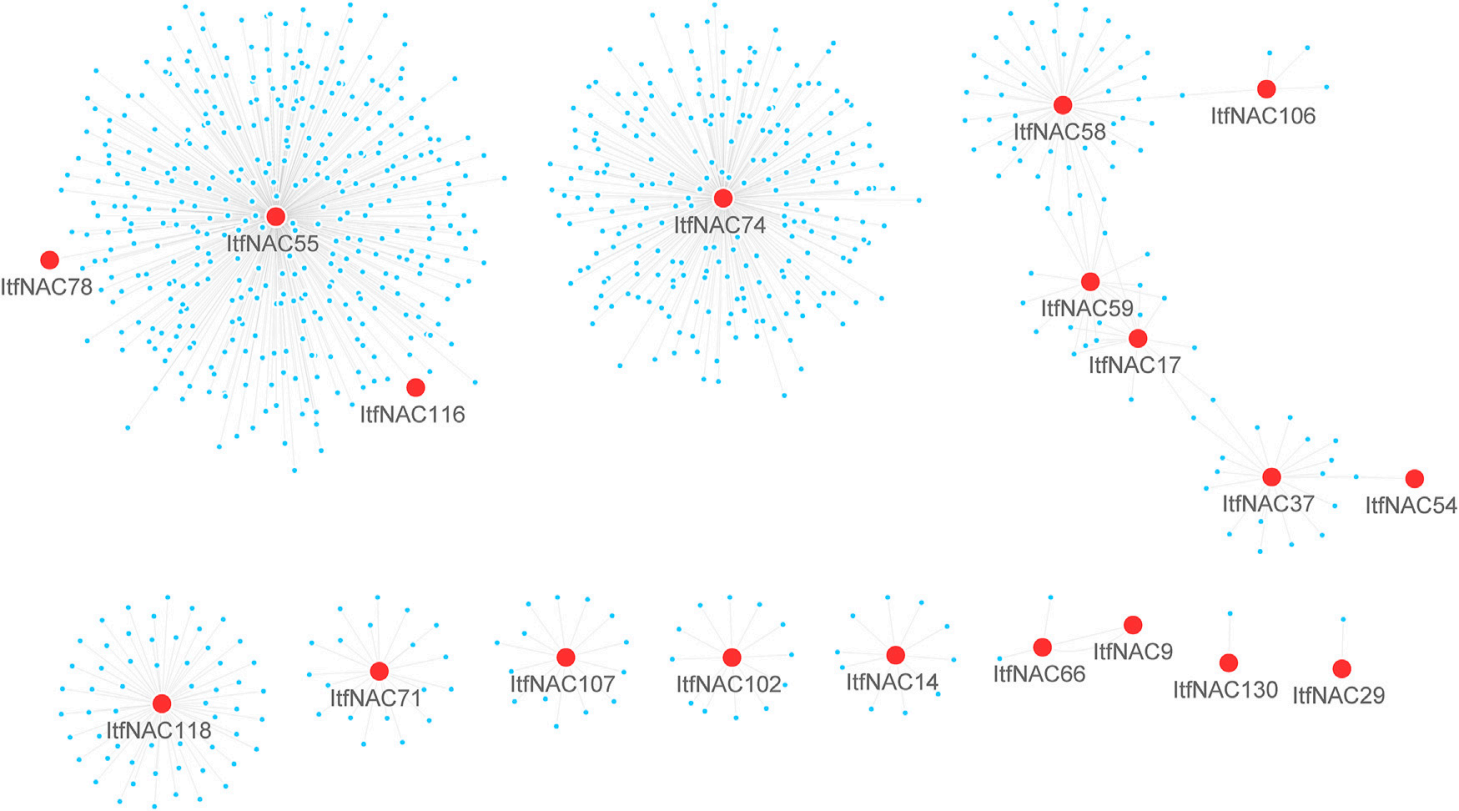
Interaction networks of ItfNAC genes. The red circle indicates NAC family proteins, and blue circle indicates other proteins.

TFs usually cooperate with other proteins to regulate the transcription of downstream target genes, so it is of fundamental importance to identify protein-protein interaction and regulation networks for NAC TFs. Using RNA-Seq data, NAC protein-protein interaction and regulation networks in the two diploid wild sweet potato species were generated by a co-expression analysis. The 11 ItbNAC proteins formed homodimers and heterodimers with each other, or interacted with other proteins. For instance, ItbNAC44 interacted with 461 other proteins including three NAC proteins (ItbNAC50, ItbNAC75, and ItbNAC110), followed by ItbNAC110, which interacted with 445 proteins including three NAC proteins (ItbNAC44, ItbNAC50 and ItbNAC75) (Figure 5, Supplementary Material S2). In *I. trifida*, 19 ItfNAC proteins interacted with at least one other protein, in particular, ItfNAC55 interacted with 387 other proteins including two NAC proteins (ItfNAC78 and ItfNAC116), and ItfNAC74 interacted with 232 other proteins (Figure 6, Supplementary Material S3).

### Tissue Expression Patterns of Identified NAC Genes in Diploid Wild Relatives of Cultivated Sweet Potato

**FIGURE 7:**
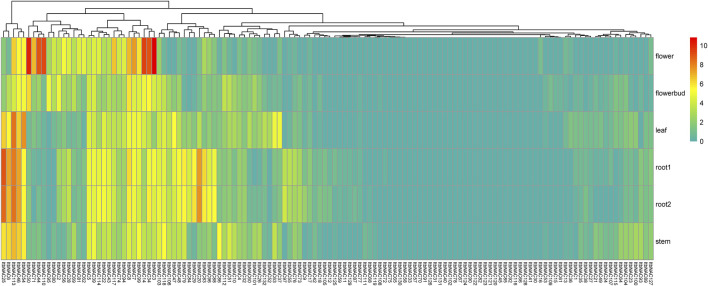
Expression patterns of ItbNAC genes in five tissues (flowers, flower buds, leaves, roots and stems) in the wild sweet potato Ipomoea triloba. The transcript abundance in different tissues was examined using RNA-Seq gene expression data. Expression profiles via a heat map were calculated from the log2 (FPKM+1) value, and shown as a blue-yellow-red gradient by using the heat map package in R version 3.4.0.

To understand tissue-related expression profiles and explore tissue-specific expression patterns, the transcript abundance of 130 *ItbNAC* genes and 144 *ItfNAC* genes in five tissues (flowers, flower buds, leaves, roots and stems) were examined using the RNA-Seq gene expression data downloaded from the Sweetpotato Genomics Resource. The results show that 28 out of 130 *ItbNAC* genes were not expressed in all five tissues, while 30 *ItbNAC* genes in flowers, 27 *ItbNAC* genes in flower buds, 31 *ItbNAC* genes in leaves, 34 *ItbNAC* genes in stems, and at least 35 *ItbNAC* genes in roots showed a high level of transcript accumulation (FPKM >5). Some tissue-specific *ItbNAC* genes were also found, such as 11 *ItbNAC* genes (*ItbNAC3*, *ItbNAC14*, *ItbNAC34*, *ItbNAC44*, *ItbNAC50*, *ItbNAC56*, *ItbNAC62*, *ItbNAC71*, *ItbNAC81*, *ItbNAC110*, *ItbNAC117*) in flowers, nine genes (*ItbNAC20*, *ItbNAC29*, *ItbNAC55*, *ItbNAC79*, *ItbNAC83*, *ItbNAC84*, *ItbNAC88*, *ItbNAC96*, *ItbNAC97*) in roots, *ItbNAC95* in flower buds, *ItbNAC37* and *ItbNAC54* in leaves, as well as *ItbNAC51* and *ItbNAC86* in stems (Figure 7, Supplementary Material S4).

**FIGURE 8:**
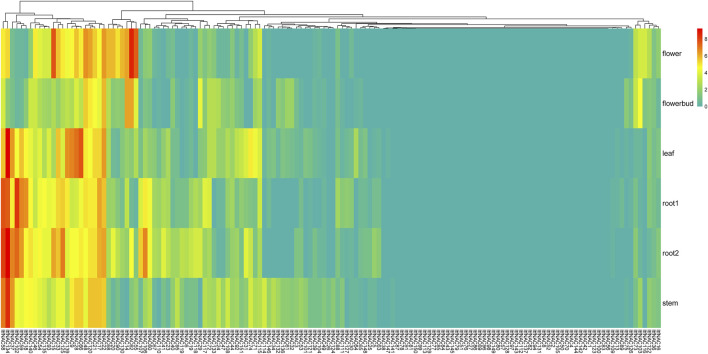
Expression patterns of ItfNAC genes in five tissues (flowers, flower buds, leaves, roots and stems) in the wild sweet potato Ipomoea trifida. The transcript abundance in different tissues was examined using RNA-Seq gene expression data. Expression profiles via a heat map were calculated from the log2 (FPKM+1) value, and shown as a blue-yellow-red gradient by using the heat map package in R version 3.4.0.

In *I. trifida*, a total of 44 *ItfNAC* genes (44/144) were not expressed in all five tissues, whereas 31 *ItfNAC* genes in flowers, 25 *ItfNAC* genes in flower buds, 33 *ItfNAC* genes in leaves and stems, and at least 32 *ItfNAC* genes in roots were highly expressed (FPKM >5). Some tissue-specific *NAC* genes were also found in 144 *ItfNACs*, such as eight genes (*ItfNAC15*, *ItfNAC20*, *ItfNAC55*, *ItfNAC62*, *ItfNAC68*, *ItfNAC90*, *ItfNAC116*, *ItfNAC130*) in flowers, five genes (*ItfNAC19*, *ItfNAC60*, *ItfNAC102*, *ItfNAC106*, *ItfNAC107*) in roots, in addition to *ItfNAC66* and *ItfNAC151* in leaves (Figure 8, Supplementary Material S5).

### Expression Profiles of Identified *ItfNAC* and *ItbNAC* Genes Under Various Stresses

**FIGURE 9:**
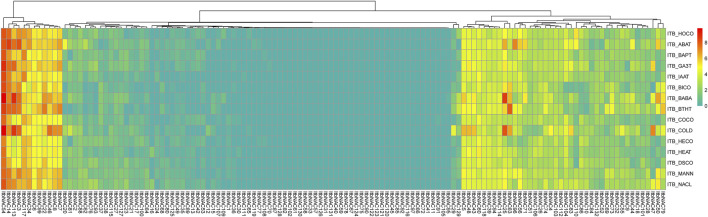
Expression profiles of ItbNAC genes under various stresses in wild sweet potato Ipomoea triloba. The transcript abundance was examined using RNA-Seq gene expression data. The expression profiles were calculated via a heat map from the log2 (FPKM+1) value, and shown as a blue-yellow-red gradient by using the heat map package in R version 3.4.0. HOCO, hormone control; ABAT, ABA treatment; BAPT, BA treatment; GA_3_T, GA_3_ treatment; IAAT, IAA treatment; BICO, biotic stress control; BABA, beta-aminobutyric acid treatment; BTHT, benzothiadiazole S-methylester treatment; COCO cold control; HECO, heat control; DSCO, drought and salt control; MANN, mannitol drought stress.

To investigate the roles of NAC TFs in response to various stresses, the transcript patterns of 130 *ItbNAC* genes and 144 *ItfNAC* genes under biotic and abiotic stresses were also analyzed using RNA-Seq gene expression data downloaded from the Sweetpotato Genomics Resource. As shown in Figure 9, 39, 15, 32, and 10 *ItbNAC* genes were upregulated in response to ABA, BA, GA_3_ and IAA treatments (FPKM >1.5-fold relative to the control). Among the 130 *ItbNAC* genes, 44 and 41 were obviously induced by BABA and BITH stress. In addition, 45, 16, 37, and 39 *ItbNAC* genes were upregulated by four abiotic stresses (cold, heat, mannitol drought and NaCl, respectively; FPKM >1.5-fold relative to the control). However, a total of 28 *ItbNAC* genes were not expressed in any sample before or after the 10 stress treatments (Supplementary Material S6).

**FIGURE 10:**
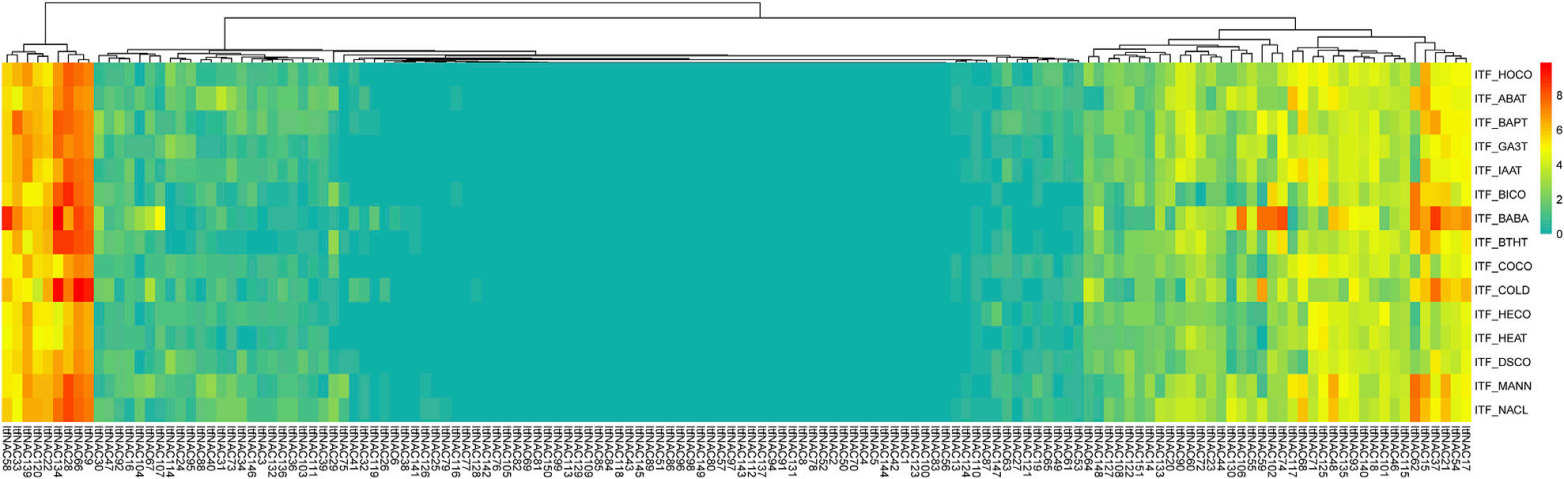
Expression profiles of ItfNAC genes under various stresses in wild sweet potato Ipomoea trifida. The transcript abundance was examined using RNA-Seq gene expression data. The expression profiles were calculated via a heat map from the log2 (FPKM+1) value, and shown as a blue-yellow-red gradient by using the heat map package in R version 3.4.0. HOCO, hormone control; ABAT, ABA treatment; BAPT, BA treatment; GA_3_T, GA_3_ treatment; IAAT, IAA treatment; BICO, biotic stress control; BABA, beta-aminobutyric acid treatment; BTHT, benzothiadiazole S-methylester treatment; COCO cold control; HECO, heat control; DSCO, drought and salt control; MANN, mannitol drought stress.

In *I. trifida* (Figure 10), upregulated genes were also selected under the various stresses with FPKM >1.5-fold when compared with each control: 33, 27, 19, and 11 *ItfNAC* genes were upregulated in response to ABA, BA, GA_3_ and IAA, respectively while 40 and 24 *ItfNAC* genes were visibly induced by BABA and BITH. Moreover, 29, 16, 34, and 39 *ItfNAC* genes were upregulated by abiotic stresses (cold, heat, mannitol drought and NaCl, respectively) whereas 41 *ItfNAC* genes were not expressed before or after any of the 10 stress treatments (Supplementary Material S7).

### Identification of Key NAC Genes Involved in Drought Stress in Cultivated Sweet Potato

**FIGURE 11:**
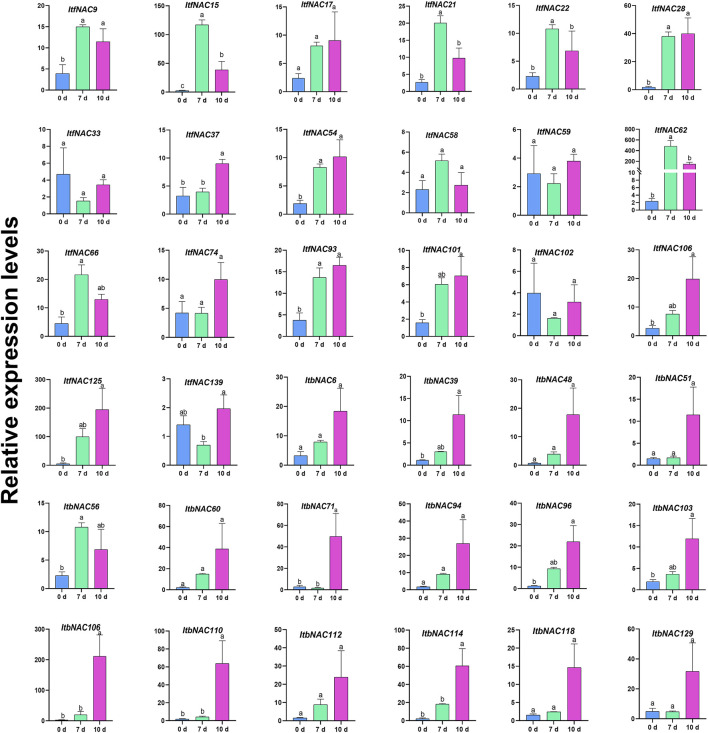
RT-qPCR expression analysis of 36 selected NAC TFs in hexaploid cultivated sweet potato plants subjected to drought stress for 0, 7, and 10 days. Three independent biological replicates and three technical replicates for each biological replicate were carried out in RT-qPCR experiments. Different letters denote significance at *p* < 0.05 using Duncan’s multiple range test.

To further investigate the key sweet potato *NAC* genes involved in drought stress, a total of 36 identified NAC TFs, including 16 *ItbNAC* genes and 20 *ItfNAC* genes that showed a higher expression level in the five tissues or in mannitol treatment, were selected for further RT-qPCR analysis. As shown in Figure 11, 21 selected NAC TFs, including eight *ItbNAC* and 13 *ItfNAC* genes, were significantly induced after 7 or 10 days of drought treatment, while the remaining 15 genes displayed no significant change in expression. The expression patterns of 26 NAC TFs (72.22% of total), including 12 *ItbNAC* and 14 *ItfNAC* genes, were consistent between drought stress and mannitol treatments. Five NAC TFs (*ItbNAC110*, *ItbNAC114*, *ItfNAC15*, *ItfNAC28*, and *ItfNAC62*), which were upregulated by mannitol treatment with FPKM >2.2-fold, were also significantly induced after 7 or 10 days of drought stress with >20-fold increase in expression when determined by RT-qPCR. A noteworthy finding is that the expression of *ItfNAC62* increased by 202.21- and 62.18-fold after 7 and 10 days of drought stress, respectively when compared with the 0-days control, so this gene deserves further functional elucidation under drought stress in sweet potato.

### 3D Structural Comparison Between ANAC019 and ItfNAC62

**FIGURE 12:**
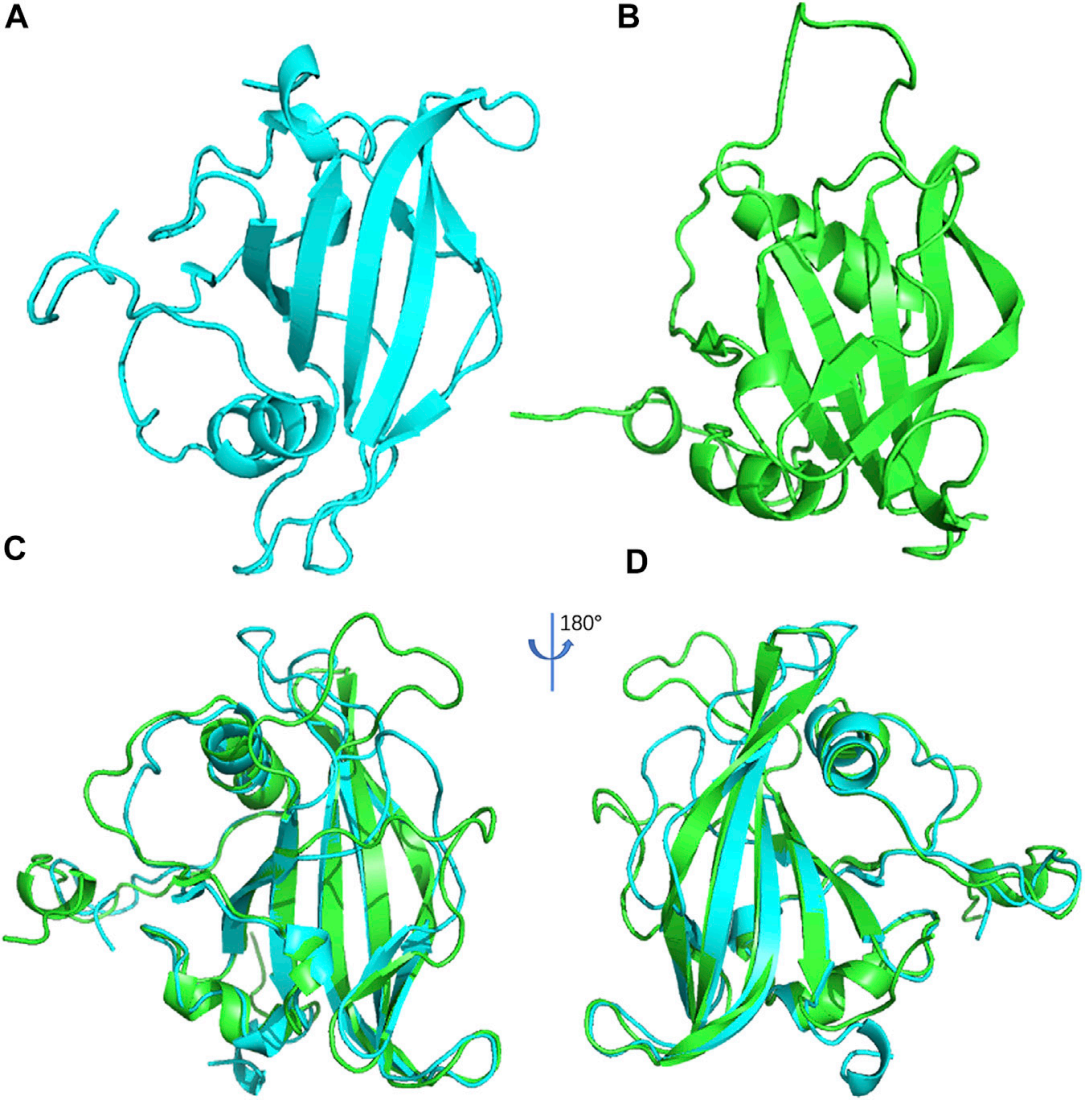
The 3D structure of ANAC019 **(A)** and ItfNAC62 **(B)** and the superposition of protein structure for ANAC019 (blue) and ItfNAC62 (green) **(C)** and its 180° rotation **(D)**.

Spatial structure analysis can provide essential information for understanding the function of a protein. To explore the function of ItfNAC62 in greater depth, its 3D structure and that of its most closely related paralogous protein, ANAC019 in *A. thaliana*, were constructed using 1UT7.A (1.9 Å) and 3ULX.A (2.6 Å) as the template, respectively. The PROCHECK program evaluated the residues in reliable regions and allowed regions, accounting for a total of 99.3% of ANAC019 (Supplementary Figure S6) and 98.5% of ItfNAC62 (Supplementary Figure S7). ERRAT values exceeded 50 in ANAC019 (83.21) and in ItfNAC62 (74.6). The Z-values (model energy) of ANAC019 and ItfNAC62, determined by the PROSA program, were within the Z-value distribution range of known structural proteins (Supplementary Figure S8). These results collectively suggest that the constructed 3D model of ANAC019 and ItfNAC62 have good reliability and quality and could be used for further analysis. Using these findings, 3D structural alignments were conducted, showing that the ANAC019 and ItfNAC62 structures are similar, especially a large overlap in the α-helix and β-sheet regions, indicating that spatial conformation is conservative between ANAC019 and ItfNAC62 (Figure 12).

### Molecular Dynamics Simulation of ANAC019 and ItfNAC62

**FIGURE 13:**
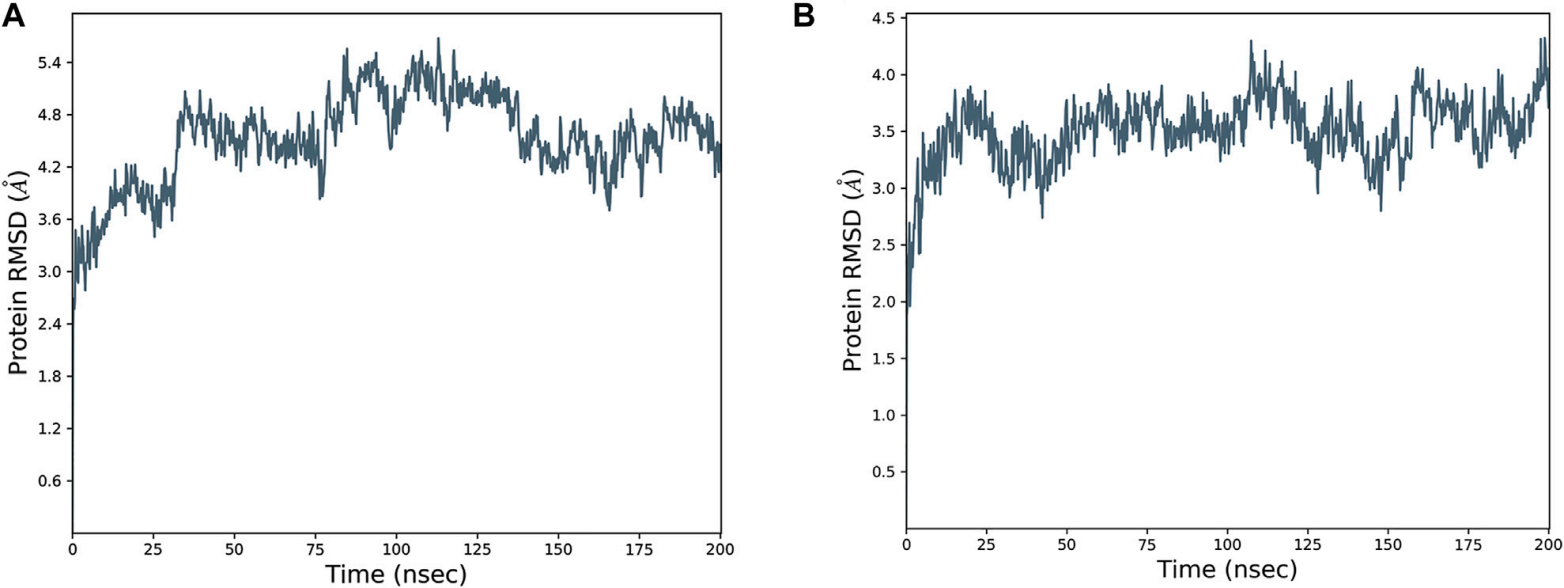
The RMSD (root-mean-square deviation) plot of ANAC019 **(A)** and ItfNAC62 **(B)** proteins for 200 ns.

In order to further verify and optimize the constructed 3D structures of ANAC019 and ItfNAC62, a 200-ns molecular dynamics simulation was performed. The RMSD value was used as an index to measure the stability of the protein structure. The RMSD value of ANAC019 gradually increased from 0 to 50 ns (0–10 ns for ItfNAC62), indicating a large change in its structure during this period, but after 50 ns (10 ns for ItfNAC62), the protein structure gradually stabilized (Figure 13). In addition, the average RMSD value of ItfNAC62 was less than that of ANAC019, suggesting that ItfNAC62 is more stable than ANAC019, mainly because of the existence of a longer and more flexible loop chain in the carbon and nitrogen end of ANAC019. In summary, the ANAC019 and ItfNAC62 structures, as assessed by homology modeling, were relatively stable, although ItfNAC62 was more stable than ANAC019.

## Discussion

NAC TFs, as one of the most abundance plant-specific transcription family genes, play a pivotal role in regulating plant organ development and providing protection against adverse environments or pathogen infection (Shao et al., 2015; Tweneboah and Oh, 2017; Yuan et al., 2019). However, detailed or complete information of NAC family genes in sweet potato had not been available, until now. Although the genome of hexaploid cultivated sweet potato was revealed (Yang et al., 2017), the incompleteness and considerable amount of redundancy and mistake in the genome assemblies caused by multiploidy genome complexity did not allow it to be used to completely or accurately identify NAC family genes in cultivated sweet potato (Wu et al., 2018). There is increasing evidence that hexaploid cultivated sweet potato arose from two diploid wild species, *I. trifida* and *I. triloba*, through allopolyploidization or autopolyploidization, or both (Kriegner et al., 2003; Muñoz-Rodriguez et al., 2018; Wu et al., 2018). Furthermore, high-quality assemblies of the *I. trifida* and *I. triloba* genomes provided robust genomic information to explore and identify NAC TF family genes for cultivated sweet potato (Wu et al., 2018). On this basis, we identified a total of 274 high confidence NAC TFs using the *I. trifida* and *I. triloba* genomes, including 130 ItbNAC and 144 ItfNAC proteins, respectively. The higher number of *NAC* genes identified in *I. trifida* than in *I. triloba* is consistent with the larger high-quality assemblies for *I. trifida* (462.0 Mb) than for *I. triloba* (457.8 Mb) (Wu et al., 2018). All 274 NAC proteins were divided into 20 subgroups based on their sequence homology and classification relative to that of *Arabidopsis* (Ooka et al., 2003). AtNAC047 was classified into the NAP group. This was different to the classification by Ooka et al. (2003), likely caused by the very close relationship between the NAP and AtNAC3 groups.

Genome duplication events, which contribute to selection pressure and provide a stronger ability for organisms to survive under diverse environmental stresses, are recognized as a main factor in the evolution and diversification of angiosperms (Crow et al., 2006). Previous reports illustrated that NAC TFs in 160 plant species evolved from common ancestors and experienced an abundance of duplicated events, which gave rise to an increase in their structural complexity, genetic variation and mutational robustness, decreased probability of extinction, as well as strengthened tolerance against changing environmental conditions (Mohanta et al., 2020). In terms of the diploid wild sweet potato in this study, 21 and 41 *NAC* genes resulted from 9 to 15 tandem duplication events in *I. triloba* and *I. trifida*, respectively (Figures 2, 3). Thus, we speculate that tandem duplication events increased the rate of evolution and expansion of these important genes, which might provide genetic variability in sweet potato species and play an important role in surviving diverse environmental stresses. Whether other duplication events such as segmental duplication or whole-genome duplication exist, were not identified in our work, but merit further research.

Gene expression pattern analysis can provide crucial clues to determine gene function (Kusano et al., 2005; Sun et al., 2018; Zhang et al., 2018). Several reports have indicated that some *NAC* genes specifically expressed in particular tissues play a vital role in regulating their development, such as *SlNAC4* in tomato which promoted fruit ripening and carotenoid accumulation (Zhu et al., 2014), and a NAC domain TF XVP, which was involved in vascular stem cell maintenance in *A. thaliana* (Yang et al., 2019). The expression profiles of the 274 *NAC* genes that were identified were examined in five tissues (flowers, flower buds, leaves, roots and stems) in two diploid sweet potatoes using RNA-Seq data. Our results showed specific and exclusive tissue expression: 11 *ItbNAC* and eight *ItfNAC* genes, mostly belonging to S7 and S14 subgroups, in flowers, 14 *NAC* genes, including nine *ItbNAC* and five *ItfNAC* genes, mainly from subgroups S7 and S9, in roots, *ItbNAC95* in flower buds, *ItbNAC37*, *ItbNAC54*, *ItfNAC66* and *ItfNAC151* in leaves, and *ItbNAC51* and *ItbNAC86* in stems (Figures 7, 8) (Supplementary Material S3, S4). Some members of group S7, which were involved in regulating tissue development, were also found in *A. thaliana*. For instance, *ANAC046* and *ANAC087* in subgroup 7 redundantly controls the onset of cell death execution in the columella root cap (Huysmans et al., 2018). *ANAC092*, another member of subgroup 7, not only negatively affected root development by regulating the expression of *ARF8* and *PIN4* through the auxin pathway, but also contributed to flower initiation (Al-Daoud and Cameron, 2011; Xi et al., 2019). Thus, we speculate that these *NAC* genes in the two diploid wild sweet potato species with tissue-specific expression may have important regulatory functions in the associated tissues, providing insight into their utilization in improving the growth and development of those tissues.

The growth of sweet potato is frequently threatened by drought stress in the field, and this stress will inevitably affect its development and cause the loss of storage root yield. To explore potential drought-resistant *NAC* genes that could be used as genetic resources in molecular breeding to generate drought-tolerant species, 36 *NAC* genes that showed higher expression levels in five tissues (flowers, flower buds, leaves, roots and stems), or after exposure to mannitol, were selected for further RT-qPCR analysis under drought stress in the hexaploid cultivated sweet potato cultivar, G8. The expression trends of most NAC TFs tested were consistent between drought stress and mannitol treatment. Five NAC TFs (*ItbNAC110*, *ItbNAC114*, *ItfNAC15*, *ItfNAC28*, and *ItfNAC62*) were simultaneously upregulated by mannitol treatment with FPKM >2.2-fold and drought stress with an increase in expression >20-fold, suggesting that all five genes may have an essential function in regulating drought or mannitol stress tolerance in sweet potato. Among them, *ItfNAC62* had the highest expression level either in response to mannitol treatment (FPKM >44-fold) or drought stress (202.21- and 62.18-fold after 7 and 10 days of drought stress, respectively). In order to further explore whether *ItfNAC62* is involved in the response to drought, a 3D structure analysis and comparison were conducted. The results showed that *ItfNAC62* had a similar spatial conformation with a paralogous *A. thaliana* gene *ANAC019*. It is widely accepted that proteins with a similar spatial structure usually have analogical functions (Najmanovich et al., 2005; Smaili et al., 2021), so we speculate that *ItfNAC62* has similar functions to *ANAC019*, which is involved in drought and biotic defense responses, which are dependent on the ABA or MeJA signaling pathway (Bu et al., 2008; Jiang et al., 2014; Li et al., 2014; Sukiran et al., 2019). ABA and MeJA response CAEs were found in the *ItfNAC62* promoter, suggesting that this gene participates in drought resistance, via the ABA or MeJA signaling pathway. Moreover, *ItfNAC62* was specifically expressed in sweet potato flowers. Its ortholog *ANAC019* also acts as an upstream regulator in promoting *A. thaliana* flower development (Sukiran et al., 2019). All this evidence suggests that there is a conservative function among evolutionarily closely homologous genes. Thus, it is reasonable to speculate that *ItfNAC62* may synergistically promote both drought resistance and flower development in sweet potato, although the putative function and mechanism need to be further investigated.

Diploid wild sweet potato *I. triloba* and *I. trifida* have the potential to improve sweet potato resilience in dry environments and should be used in introgression breeding of new sweet potato species (Guerrero-Zurita et al., 2020). A somatic hybrid sweet potato species KT1, which was obtained through protoplast fusion between sweet potato (*I. batatas*.) cv. Kokei No. 14 and its wild relative *I. triloba*, exhibited significantly higher drought tolerance than Kokei No. 14 under drought stress through a significant increase in the content of proline, activities of superoxide dismutase and photosynthesis, but decreased malonaldehyde content (Jia et al., 2017). These studies suggest that diploid wild sweet potato could provide prominent gene resources to improve the tolerance of cultivated sweet potato against adverse environments. Thus, the *NAC* genes selected from two diploid wild sweet potato species in this study could be used as promising candidates for genetic engineering to create new sweet potato germplasm with broad-spectrum tolerance, especially drought resistance against adverse conditions.

## Conclusion

In this study, we identified 274 high confidence NAC domain TFs in the genomes of two diploid wild sweet potato species. Those TFs could be divided into 20 subgroups based on a phylogenetic analysis, including *NAC* family genes from *A. thaliana*. Gene motifs, structure, CAEs and interaction networks were carefully analyzed to thoroughly understand the diversity and interaction of the identified *NAC* genes. Gene chromosomal location and synteny analysis showed that tandem duplication events occurred in both diploid wild sweet potato genomes. In addition, expression pattern analysis in five tissues and under various stresses revealed some tissue-specific expression and significantly upregulated stimuli response NAC genes. Our study provides comprehensive and systematic information about *NAC* genes in two diploid wild sweet potato species. Some selected genes upregulated by drought may lay a foundation for sweet potato drought-tolerance breeding.

## Data Availability

The datasets presented in this study can be found in online repositories. The names of the repository/repositories and accession number(s) can be found in the article/Supplementary Material.
