# Supplementary figures and images for: Genome-Wide Identification and Analysis of NAC Transcription Factor Family in Two Diploid Wild Relatives of Cultivated Sweet Potato Uncovers Potential NAC Genes Related to Drought Tolerance
Source: Front Genet. 2021 Nov 24;12:744220. doi: 10.3389/fgene.2021.744220 (PMC8653416; doi:10.3389/fgene.2021.744220)

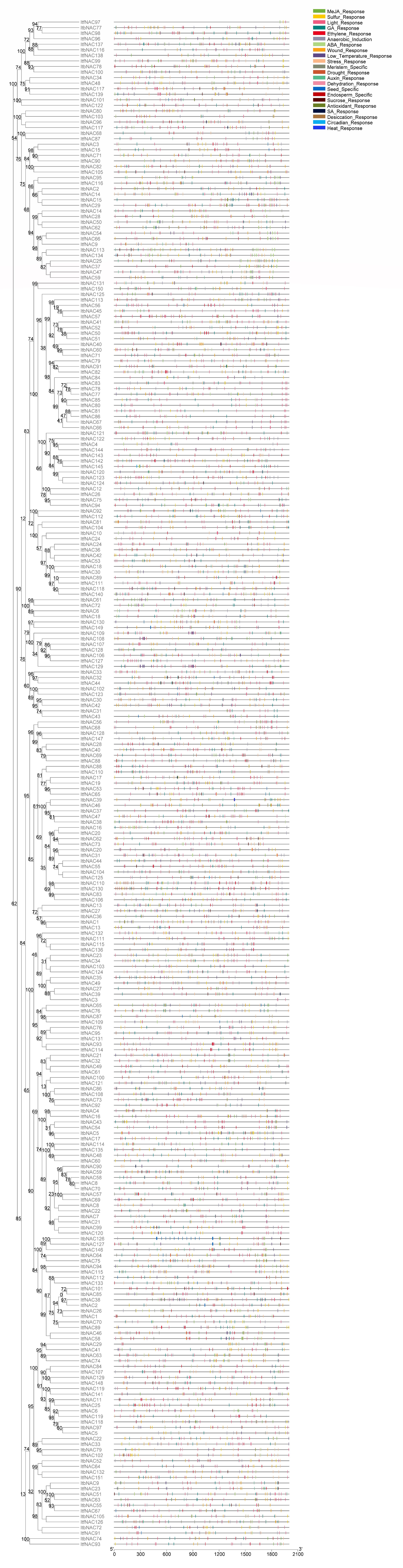

Supplement: Supplementary file 1 [file Image3.JPEG]

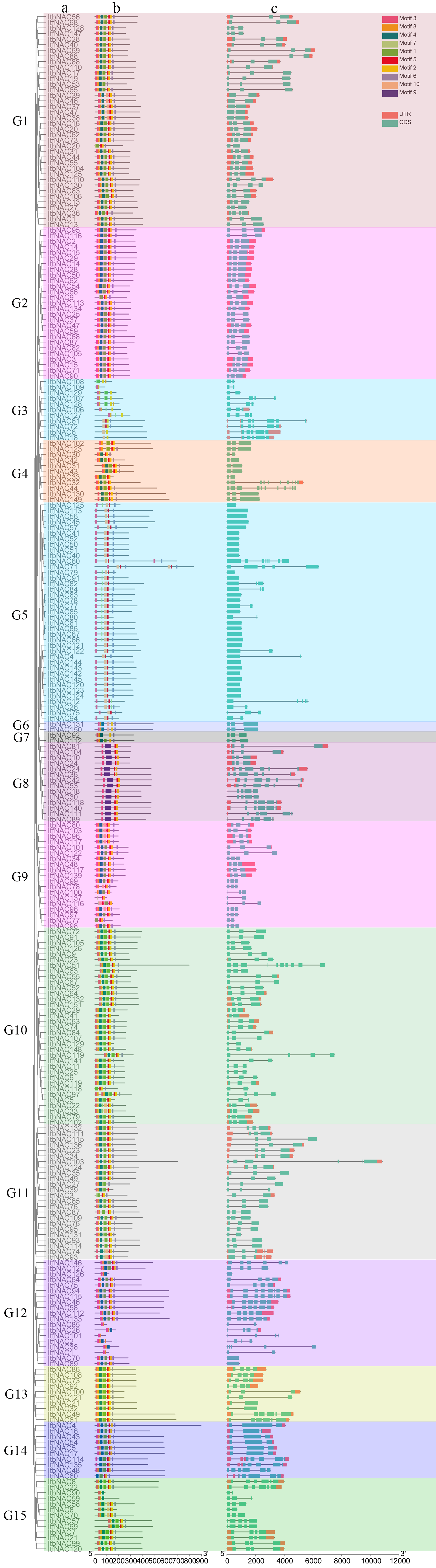

Supplement: Supplementary file 4 [file Image2.TIF]

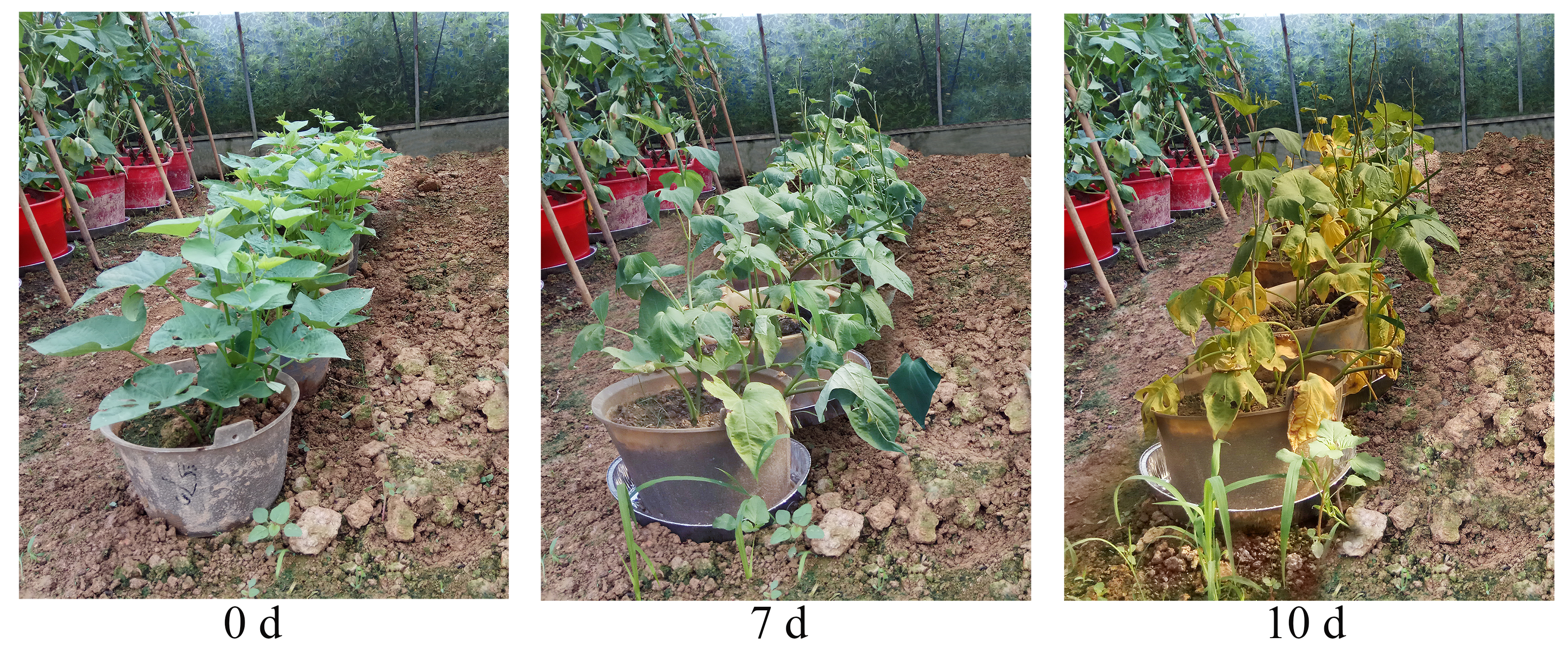

Supplement: Supplementary file 5 [file Image1.TIF]
