# Supplementary material for: Genome-Wide Identification and Analysis of NAC Transcription Factor Family in Two Diploid Wild Relatives of Cultivated Sweet Potato Uncovers Potential NAC Genes Related to Drought Tolerance
Source: Front Genet. 2021 Nov 24;12:744220. doi: 10.3389/fgene.2021.744220 (PMC8653416; doi:10.3389/fgene.2021.744220)
Supplement: Supplementary file 2 [file Table1.PDF]

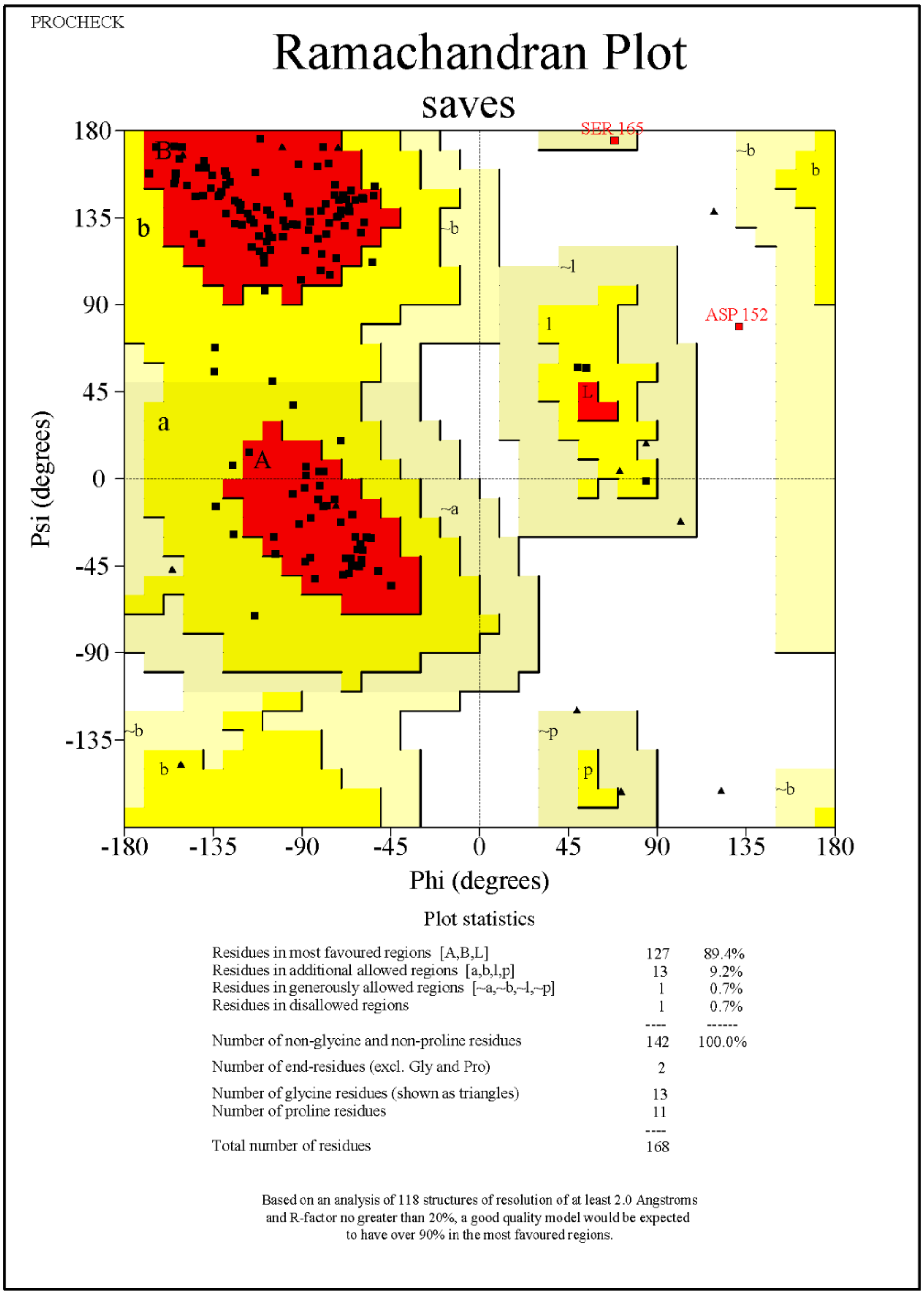


Supplementary Figure S6: Ramachandran of ANAC019

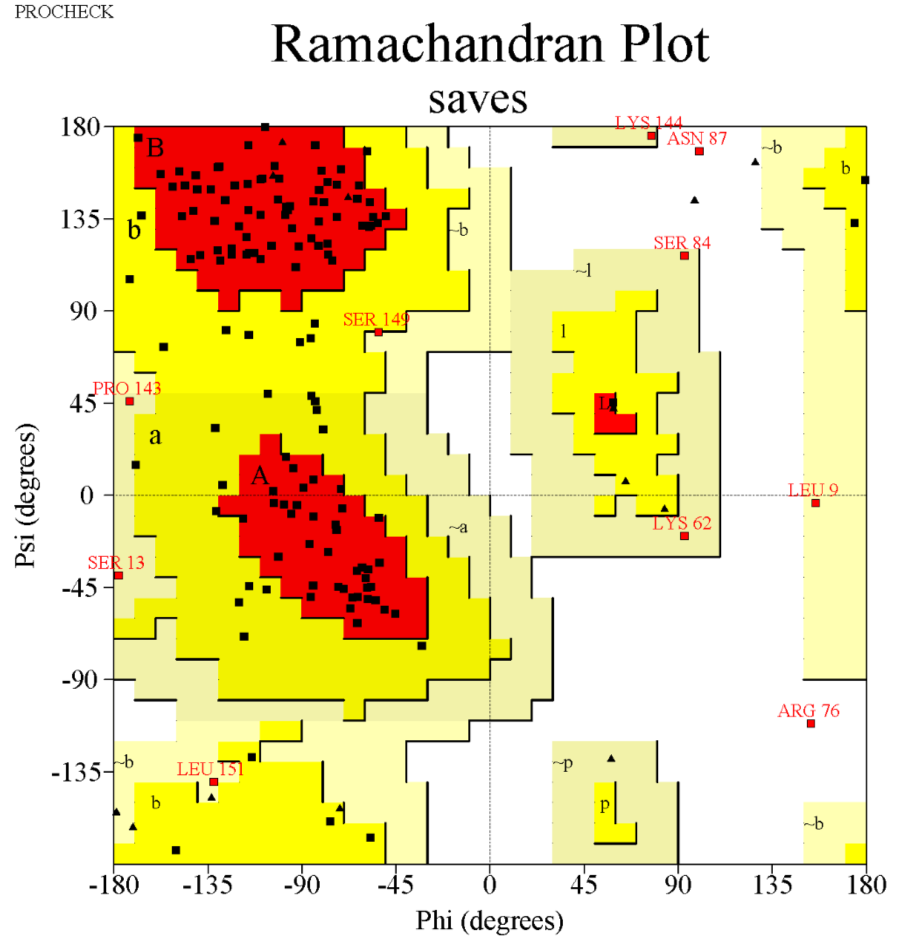


Plot statistics

| | | |
|---|---|---|
| Residues in most favoured regions [A,B,L] | 99 | 72.3% |
| Residues in additional allowed regions [a,b,l,p] | 29 | 21.2% |
| Residues in generously allowed regions [~a,~b,~l,~p] | 7 | 5.1% |
| Residues in disallowed regions | 2 | 1.5% |
| | ---- | ------ |
| Number of non-glycine and non-proline residues | 137 | 100.0% |
| Number of end-residues (excl. Gly and Pro) | 1 | |
| Number of glycine residues (shown as triangles) | 13 | |
| Number of proline residues | 11 | |
| | ---- | |
| Total number of residues | 162 | |

Based on an analysis of 118 structures of resolution of at least 2.0 Angstroms and R-factor no greater than 20%, a good quality model would be expected to have over 90% in the most favoured regions.

Supplementary Figure S7: Ramachandran of ItfNAC62

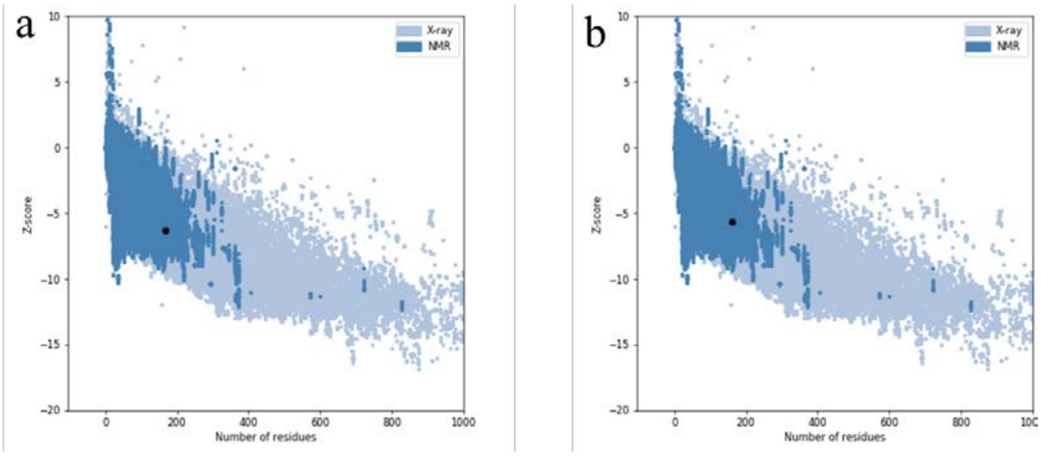


Supplementary Figure S8: PROSA results for ANAC019 (a) and ItfNAC62 (b). All of the experiments in the PDB database determined the Z value of the protein structure chain which is similar in size with the target model, and formed a distribution area. If the Z value of the target model falls into this distribution, the model structure is reasonable. The black dots indicate the location of the constructed proteins, that is, the ANAC019 and ItfNAC62 fall within the Z value distribution range of the known structural protein, so the ANAC019 and ItfNAC62 structures are reasonable.
