# Supplementary material for: Genome-Wide Identification and Analysis of NAC Transcription Factor Family in Two Diploid Wild Relatives of Cultivated Sweet Potato Uncovers Potential NAC Genes Related to Drought Tolerance
Source: Front Genet. 2021 Nov 24;12:744220. doi: 10.3389/fgene.2021.744220 (PMC8653416; doi:10.3389/fgene.2021.744220)
Supplement: Supplementary file 6 [file Table2.DOCX]

Table S1: RT-qPCR primer used in this study.

| Gene name | Forward/Reverse primer | Forward/Reverse primer sequence | Fragment size (bp) |
| --- | --- | --- | --- |
| GAPDH | GAPDH-F | AGGATGCCCCAATGTTTGTT | 180 |
|  | GAPDH-R | TCTGAGTAGCGGTGATGGAGTG |  |
| ItfNAC9 | ItfNAC9-F | AGCATCCGAAATATGAGCAAAG | 131 |
|  | ItfNAC9-R | AGGTACAGGAAATCGCTGAAAA |  |
| ItfNAC15 | ItfNAC15-F | TGCCCTGTCTCCATTATCCC | 190 |
|  | ItfNAC15-R | TGGCTTTGTCTGTGCCTGTG |  |
| ItfNAC17 | ItfNAC17-F | GTCATCATCGGCTTCCCTTTC | 207 |
|  | ItfNAC17-R | CATTGCCTGTCGCCAGTTTT |  |
| ItfNAC21 | ItfNAC21-F | TCGCCCTGTCCGTCATAAGT | 104 |
|  | ItfNAC21-R | CGTGCATAACCCAATTCGTC |  |
| ItfNAC22 | ItfNAC22-F | AGGGCTGTCAATCATCGGTCTA | 248 |
|  | ItfNAC22-R | TCCTCCCACTCTTCCTCCAC |  |
| ItfNAC28 | ItfNAC28-F | AGATAATAACCACCGAAGGACG | 166 |
|  | ItfNAC28-R | CATAGCACCCATTCATCAAGC |  |
| ItfNAC33 | ItfNAC33-F | GTCGGAAGGGAGGAAAGTGTT | 230 |
|  | ItfNAC33-R | CGGCTACTGCGTATCCTTGTT |  |
| ItfNAC37 | ItfNAC37-F | GCAGTTGGACAGATCGGAGTC | 172 |
|  | ItfNAC37-R | GAAAGGAAAGGTGGTTTGAAGG |  |
| ItfNAC54 | ItfNAC54-F | ATGTTATCGGCGAGACTGACG | 159 |
|  | ItfNAC54-R | AGTAGCCATGTTTGGTTGCTCT |  |
| ItfNAC58 | ItfNAC58-F | CAGAGCGAGGGTACTGGAAG | 204 |
|  | ItfNAC58-R | AAAGCACAAAAGGAGCCTGA |  |
| ItfNAC59 | ItfNAC59-F | CTCAGGCTTGATGACTGGGTAC | 200 |
|  | ItfNAC59-R | ATTTGGTCGGAGGAGGAGC |  |
| ItfNAC62 | ItfNAC62-F | TGTGCCGAATTTACAAGAAGC | 155 |
|  | ItfNAC62-R | CACGAAAGACTGGTCGTTGAG |  |
| ItfNAC66 | ItfNAC66-F | GCATCCGAAATATGAGCAAACC | 120 |
|  | ItfNAC66-R | AATCGCTGAACACCTGTGGC |  |
| ItfNAC74 | ItfNAC74-F | CCCAAGCTACGTCTACTCATCG | 210 |
|  | ItfNAC74-R | CGGCACAGGGTCATTTCAT |  |
| ItfNAC93 | ItfNAC93-F | GAGGGAGGGATAGGGTTGTTGT | 205 |
|  | ItfNAC93-R | TTTCCGTGATGGGATTTGG |  |
| ItfNAC101 | ItfNAC101-F | ATCAGAAAGATCGACGAAGACG | 178 |
|  | ItfNAC101-R | CTCCAATGAGAACCTCCCATAA |  |
| ItfNAC102 | ItfNAC102-F | CTTGGGACTTGCCAGGACAT | 220 |
|  | ItfNAC102-R | GCTCTGCCTTGATAGAACACCA |  |
| ItfNAC106 | ItfNAC106-F | CAACGACGCCGTATCAAGGA | 102 |
|  | ItfNAC106-R | TCGGAGTAATCTGGGAAATCG |  |
| ItfNAC125 | ItfNAC125-F2 | AGAGCACCCAGAGGTGAGAA | 170 |
|  | ItfNAC125-R2 | TTTTCACCTCCGATCAGACC |  |

Table S1 continued.

| Gene name | Forward/Reverse primer | Forward/Reverse primer sequence | Fragment size |
| --- | --- | --- | --- |
| ItfNAC139 | ItfNAC139-F | CGGATGAGGAGTTGGTGGTT | 199 |
|  | ItfNAC139-R | GAGCTGGTGGCTCTGTTTGAC |  |
| ItbNAC6 | ItbNAC6-F | CAGGAGTACAAGACCGAGAAGG | 178 |
|  | ItbNAC6-R | AAGTAGGGTTAGCATTGGGAGC |  |
| ItbNAC39 | ItbNAC39-F | GAGGGCCACAATTCCTTGC | 228 |
|  | ItbNAC39-R | CTGCCTCACTCCTCCAACATAC |  |
| ItbNAC48 | ItbNAC48-F | TCAGTGAGGAAGACTGGGAAGA | 210 |
|  | ItbNAC48-R | GTTTGGTAGGAGGAGTGGCATA |  |
| ItbNAC51 | ItbNAC51-F | TGGGCAGGAGTGAGTCGTCA | 201 |
|  | ItbNAC51-R | TGTTCGGAACTTGGCATCG |  |
| ItbNAC56 | ItbNAC56-F | GTGCGAGCCTTGGGACATT | 218 |
|  | ItbNAC56-R | TTGGAGCCCTTCCTTGGTAA |  |
| ItbNAC60 | ItbNAC60-F | GCTCCTACATCAGTTGTGGCG | 201 |
|  | ItbNAC60-R | CGTAGCCCTGGACTTCAAACA |  |
| ItbNAC71 | ItbNAC71-F | CCAAACGGCATTCGTCCTA | 135 |
|  | ItbNAC71-R | AGGTGGCTTGCCTTGATAGAA |  |
| ItbNAC94 | ItbNAC94-F2 | TAGGTGCAACAGAGCCACAG | 250 |
|  | ItbNAC94-R2 | GCGACAAAGGACAAAAGCTC |  |
| ItbNAC96 | ItbNAC96-F | CCGATTATTCCGCTTCCACT | 121 |
|  | ItbNAC96-R | CCGCCACCGTTATCATAGTTT |  |
| ItbNAC103 | ItbNAC103-F2 | CCCTTCATTTGCAAGAGAGC | 111 |
|  | ItbNAC103-R2 | GATGCATCTGCTGCTGGTAA |  |
| ItbNAC106 | ItbNAC106-F | AGTATGCGAGGAACTATGAGGC | 249 |
|  | ItbNAC106-R | TCTGAAGATAATGGGCAGGAAC |  |
| ItbNAC110 | ItbNAC110-F2 | GGAAGGCTACCGGAAAAGAC | 242 |
|  | ItbNAC110-R2 | CTCCATTGGGATTCCTCTCA |  |
| ItbNAC112 | ItbNAC112-F | GGACCTGCCTGATTTGTCTATG | 125 |
|  | ItbNAC112-R | CCTTCCAGTACCCTCGCTCT |  |
| ItbNAC114 | ItbNAC114-F | CAATGTTCGTCTTTACCACCCA | 129 |
|  | ItbNAC114-R | GTCTCAGTTGCCCGCTTCA |  |
| ItbNAC118 | ItbNAC118-F | CAGCAACGGGAAGTGGATT | 133 |
|  | ItbNAC118-R | GCTCCTCACCACCTTCATTCT |  |
| ItbNAC129 | ItbNAC129-F2 | CCCGACAGAAGAAGAACTGG | 177 |
|  | ItbNAC129-R2 | TGCATTGGGATGAAGAAAAA |  |

Table S2: The confidence level and characteristics of identified *ItbNACs*.

| Gene  Name | ID | Confidence level | ORF  (aa) | PI | Molecular weight (KD) | Subcellular location |
| --- | --- | --- | --- | --- | --- | --- |
| ItbNAC1 | itb01g00360.t1 | High | 402 | 7.64 | 44.84 | Nuclear |
| ItbNAC2 | itb01g08160.t1 | High | 340 | 8.93 | 37.56 | Nuclear |
| ItbNAC3 | itb01g25540.t1 | High | 282 | 8.66 | 31.97 | Nuclear |
| ItbNAC4 | itb01g30560.t1 | High | 896 | 4.33 | 101.97 | Nuclear |
| ItbNAC5 | itb01g30570.t1 | High | 585 | 4.44 | 65.89 | Nuclear |
| ItbNAC6 | itb01g34330.t1 | High | 439 | 4.48 | 48.37 | Nuclear |
| ItbNAC7 | itb02g02010.t1 | High | 400 | 5.06 | 44.82 | Nuclear |
| ItbNAC8 | itb02g02020.t1 | High | 535 | 4.36 | 59.83 | Cytoplasmic |
| ItbNAC9 | itb02g05540.t1 | High | 283 | 8.74 | 32.83 | Nuclear |
| ItbNAC10 | itb02g08080.t1 | High | 293 | 8.79 | 32.95 | Nuclear |
| ItbNAC11 | itb02g09010.t1 | High | 248 | 6.86 | 27.75 | Nuclear |
| ItbNAC12 | itb02g09930.t1 | High | 248 | 7.86 | 29.07 | Nuclear |
| ItbNAC13 | itb02g10520.t1 | High | 356 | 8.11 | 39.4 | Chloroplast |
| ItbNAC14 | itb02g10930.t1 | High | 336 | 8.4 | 37.49 | Nuclear |
| ItbNAC15 | itb02g10940.t1 | High | 353 | 9.1 | 38.99 | Nuclear |
| ItbNAC16 | itb02g16600.t1 | High | 330 | 8.7 | 36.98 | Cytoplasmic |
| ItbNAC17 | itb02g19330.t1 | High | 326 | 6.63 | 36.83 | Nuclear |
| ItbNAC18 | itb03g00480.t1 | High | 390 | 4.94 | 43.19 | Nuclear |
| ItbNAC19 | itb03g01560.t1 | Low | - | - | - | - |
| ItbNAC20 | itb03g01570.t1 | High | 234 | 7.11 | 26.12 | Cytoplasmic |
| ItbNAC21 | itb03g04980.t1 | High | 352 | 5.99 | 40.14 | Nuclear |
| ItbNAC22 | itb03g06230.t1 | High | 258 | 8.93 | 29.31 | Cytoplasmic |
| ItbNAC23 | itb03g07070.t1 | High | 354 | 5.18 | 40.18 | Nuclear |
| ItbNAC24 | itb03g19100.t1 | High | 474 | 6.95 | 52.43 | Nuclear |
| ItbNAC25 | itb03g27700.t1 | High | 288 | 8.34 | 33.23 | Nuclear |
| ItbNAC26 | itb04g17510.t1 | High | 176 | 7.43 | 19.5 | Extracellular |
| ItbNAC27 | itb04g20480.t1 | High | 293 | 5.54 | 33.61 | Nuclear |
| ItbNAC28 | itb04g25600.t1 | High | 291 | 6.51 | 33.04 | Cytoplasmic |
| ItbNAC29 | itb04g31640.t1 | High | 274 | 8.26 | 30.48 | Nuclear |
| ItbNAC30 | itb04g32970.t1 | High | 137 | 6.78 | 16.04 | Cytoplasmic |
| ItbNAC31 | itb04g32980.t1 | High | 323 | 5.01 | 36.78 | Nuclear |
| ItbNAC32 | itb04g33390.t1 | High | 382 | 6.89 | 43.07 | Cytoplasmic |
| ItbNAC33 | itb04g33400.t1 | High | 155 | 9.6 | 18 | Cytoplasmic |
| ItbNAC34 | itb05g00330.t1 | High | 242 | 9.37 | 27.29 | Nuclear |
| ItbNAC35 | itb05g01340.t1 | High | 347 | 6.65 | 39.69 | Nuclear |
| ItbNAC36 | itb05g01640.t1 | High | 320 | 9.05 | 36.94 | Nuclear |
| ItbNAC37 | itb05g01660.t1 | High | 384 | 7.52 | 43.22 | Nuclear |
| ItbNAC38 | itb05g01690.t1 | High | 381 | 7.3 | 43.23 | Nuclear |
| ItbNAC39 | itb05g01720.t1 | High | 344 | 8.27 | 39.63 | Nuclear |
| ItbNAC40 | itb05g03890.t1 | High | 287 | 4.38 | 32.56 | Cytoplasmic |

Table S2 continued

| Gene Name | ID | Confidence  level | ORF  (aa) | PI | Molecular weight (KD) | Subcellular location |
| --- | --- | --- | --- | --- | --- | --- |
| ItbNAC41 | itb05g03930.t1 | High | 287 | 4.25 | 32.51 | Cytoplasmic |
| ItbNAC42 | itb05g07450.t1 | High | 479 | 6.61 | 53.81 | Nuclear |
| ItbNAC43 | itb05g12920.t1 | High | 578 | 4.63 | 64.56 | Nuclear |
| ItbNAC44 | itb05g16480.t1 | High | 294 | 6.42 | 33.37 | Cytoplasmic |
| ItbNAC45 | itb05g17120.t1 | High | 502 | 4.03 | 54.9 | Nuclear |
| ItbNAC46 | itb05g20680.t1 | High | 579 | 4.73 | 63.76 | Nuclear |
| ItbNAC47 | itb05g21610.t1 | High | 273 | 6.63 | 30.98 | Chloroplast |
| ItbNAC48 | itb05g21760.t1 | High | 591 | 4.7 | 65.4 | Nuclear |
| ItbNAC49 | itb05g23700.t1 | High | 677 | 6.64 | 77.34 | Nuclear |
| ItbNAC50 | itb05g26030.t1 | High | 322 | 7.21 | 35.73 | Chloroplast |
| ItbNAC51 | itb05g27860.t1 | High | 796 | 8.59 | 90.53 | Plasma Membrane |
| ItbNAC52 | itb06g11220.t1 | High | 357 | 7.24 | 40.48 | Nuclear |
| ItbNAC53 | itb06g12220.t1 | High | 267 | 4.95 | 30.09 | Nuclear |
| ItbNAC54 | itb06g12990.t1 | High | 294 | 6.26 | 33.34 | Nuclear |
| ItbNAC55 | itb06g13370.t1 | High | 301 | 7.38 | 34.73 | Nuclear |
| ItbNAC56 | itb06g13570.t1 | High | 361 | 8.13 | 39.35 | Nuclear |
| ItbNAC57 | itb06g17690.t1 | High | 481 | 4.57 | 52.95 | Cytoplasmic |
| ItbNAC58 | itb06g17700.t1 | High | 333 | 7.31 | 38.07 | Cytoplasmic |
| ItbNAC59 | itb06g17710.t1 | High | 204 | 7.74 | 22.52 | Nuclear |
| ItbNAC60 | itb06g18350.t1 | High | 693 | 4.15 | 78.62 | Plasma Membrane |
| ItbNAC61 | itb06g21020.t1 | High | 420 | 5.6 | 46.92 | Nuclear |
| ItbNAC62 | itb06g24940.t1 | High | 331 | 6.78 | 37.23 | Nuclear |
| ItbNAC63 | itb07g00590.t1 | High | 266 | 6.78 | 30.75 | Nuclear |
| ItbNAC64 | itb07g00640.t1 | High | 390 | 7.23 | 43.68 | Nuclear |
| ItbNAC65 | itb07g03390.t1 | High | 354 | 6.98 | 39.64 | Nuclear |
| ItbNAC66 | itb07g04880.t1 | High | 366 | 4.32 | 41.46 | Nuclear |
| ItbNAC67 | itb07g04890.t1 | High | 351 | 4.68 | 40.71 | Nuclear |
| ItbNAC68 | itb07g12890.t1 | High | 333 | 9.21 | 37.97 | Nuclear |
| ItbNAC69 | itb07g19430.t1 | High | 278 | 6.35 | 31.61 | Cytoplasmic |
| ItbNAC70 | itb07g19690.t1 | High | 281 | 4.9 | 32.13 | Nuclear |
| ItbNAC71 | itb07g22560.t1 | High | 294 | 8.29 | 33.36 | Nuclear |
| ItbNAC72 | itb07g22780.t1 | High | 393 | 7.49 | 43.41 | Nuclear |
| ItbNAC73 | itb08g01750.t1 | High | 348 | 5.42 | 38.97 | Nuclear |
| ItbNAC74 | itb08g04590.t1 | High | 281 | 5.72 | 31.29 | Nuclear |
| ItbNAC75 | itb08g08010.t1 | High | 228 | 10.13 | 26.15 | Mitochondrial |
| ItbNAC76 | itb08g08080.t1 | High | 314 | 6.87 | 35.51 | Nuclear |
| ItbNAC77 | itb08g11090.t1 | High | 147 | 10.39 | 16.31 | Nuclear |
| ItbNAC78 | itb08g11200.t1 | High | 179 | 9.45 | 19.99 | Extracellular |
| ItbNAC79 | itb09g00190.t1 | High | 335 | 6.57 | 37.79 | Nuclear |
| ItbNAC80 | itb09g04010.t1 | High | 198 | 4.8 | 22.36 | Nuclear |

Table S2 continued

| Gene  Name | ID | Confidence  level | ORF  (aa) | PI | Molecular weight (KD) | Subcellular location |
| --- | --- | --- | --- | --- | --- | --- |
| ItbNAC81 | itb09g07530.t1 | High | 300 | 8.24 | 33.58 | Nuclear |
| ItbNAC82 | itb09g07550.t1 | High | 267 | 8.67 | 30.31 | Nuclear |
| ItbNAC83 | itb09g11420.t1 | High | 322 | 6.79 | 35.9 | Chloroplast |
| ItbNAC84 | itb09g14300.t1 | High | 258 | 7.38 | 28.99 | Nuclear |
| ItbNAC85 | itb09g27800.t1 | High | 99 | 11.57 | 11.68 | Mitochondrial |
| ItbNAC86 | itb09g28870.t1 | High | 342 | 6.78 | 38.84 | Nuclear |
| ItbNAC87 | itb09g31150.t1 | High | 375 | 7 | 42.35 | Nuclear |
| ItbNAC88 | itb10g00650.t1 | High | 342 | 8.64 | 37.56 | Nuclear |
| ItbNAC89 | itb10g08640.t1 | High | 429 | 5.57 | 49.35 | Nuclear |
| ItbNAC90 | itb10g08710.t1 | High | 86 | 6.09 | 9.86 | Cytoplasmic |
| ItbNAC91 | itb10g12270.t1 | High | 282 | 5.3 | 32.94 | Nuclear |
| ItbNAC92 | itb10g14620.t1 | High | 325 | 5.32 | 37.98 | Nuclear |
| ItbNAC93 | itb10g16710.t1 | High | 380 | 6.66 | 42.54 | Nuclear |
| ItbNAC94 | itb10g16890.t1 | High | 622 | 6 | 68.41 | Nuclear |
| ItbNAC95 | itb10g20290.t1 | High | 349 | 8.03 | 38.27 | Nuclear |
| ItbNAC96 | itb10g22690.t1 | High | 197 | 4.83 | 22.6 | Nuclear |
| ItbNAC97 | itb11g03540.t1 | High | 308 | 4.96 | 35.05 | Nuclear |
| ItbNAC98 | itb11g03550.t1 | Low | - | - | - | - |
| ItbNAC99 | itb11g07890.t1 | High | 390 | 5.05 | 43.57 | Nuclear |
| ItbNAC100 | itb11g12500.t1 | High | 246 | 4.59 | 28.52 | Nuclear |
| ItbNAC101 | itb11g20720.t1 | High | 281 | 7.47 | 31.52 | Nuclear |
| ItbNAC102 | itb12g00300.t1 | High | 471 | 4.66 | 54.12 | Nuclear |
| ItbNAC103 | itb12g02990.t1 | High | 696 | 7.31 | 79.34 | Nuclear |
| ItbNAC104 | itb12g06800.t1 | High | 285 | 7.61 | 32.16 | Nuclear |
| ItbNAC105 | itb12g06940.t1 | High | 357 | 7.63 | 40 | Nuclear |
| ItbNAC106 | itb12g08950.t1 | High | 218 | 6.81 | 26.06 | Cytoplasmic |
| ItbNAC107 | itb12g08970.t1 | High | 237 | 6.64 | 28.23 | Cytoplasmic |
| ItbNAC108 | itb12g08980.t1 | High | 140 | 8.61 | 16.62 | Cytoplasmic |
| ItbNAC109 | itb12g08990.t1 | High | 86 | 4.07 | 10.2 | Nuclear |
| ItbNAC110 | itb12g13220.t1 | High | 375 | 4.76 | 42.19 | Cytoplasmic |
| ItbNAC111 | itb12g24950.t1 | High | 358 | 6.83 | 41.01 | Nuclear |
| ItbNAC112 | itb12g27080.t1 | High | 545 | 4.57 | 60.1 | Nuclear |
| ItbNAC113 | itb12g27850.t1 | High | 300 | 7.16 | 34.67 | Cytoplasmic |
| ItbNAC114 | itb12g28030.t1 | High | 445 | 4.46 | 48.84 | Nuclear |
| ItbNAC115 | itb13g06670.t1 | High | 338 | 7 | 39.36 | Nuclear |
| ItbNAC116 | itb13g17700.t1 | High | 151 | 11.11 | 17.4 | Mitochondrial |
| ItbNAC117 | itb13g20210.t1 | High | 256 | 8.65 | 28.73 | Nuclear |
| ItbNAC118 | itb13g26520.t1 | High | 474 | 4.8 | 53.79 | Nuclear |
| ItbNAC119 | itb14g00640.t1 | High | 324 | 6.31 | 37.13 | Nuclear |
| ItbNAC120 | itb14g05040.t1 | High | 298 | 5.14 | 33.26 | Nuclear |

Table S2 continued

| Gene  Name | ID | Confidence  level | ORF  (aa) | PI | Molecular weight (KD) | Subcellular location |
| --- | --- | --- | --- | --- | --- | --- |
| ItbNAC121 | itb14g05200.t1 | High | 345 | 4.69 | 38.95 | Nuclear |
| ItbNAC122 | itb14g05210.t1 | High | 389 | 4.88 | 43.97 | Nuclear |
| ItbNAC123 | itb14g05290.t1 | High | 323 | 4.77 | 36.36 | Nuclear |
| ItbNAC124 | itb14g05300.t1 | High | 323 | 4.77 | 36.36 | Nuclear |
| ItbNAC125 | itb14g05780.t1 | High | 213 | 7.45 | 24.98 | Nuclear |
| ItbNAC126 | itb14g08420.t1 | High | 119 | 4.17 | 13.62 | Nuclear |
| ItbNAC127 | itb14g08430.t1 | High | 425 | 7.25 | 48.39 | Nuclear |
| ItbNAC128 | itb14g19720.t1 | High | 259 | 6.39 | 29.14 | Cytoplasmic |
| ItbNAC129 | itb15g01680.t1 | High | 156 | 9.24 | 18.29 | Nuclear |
| ItbNAC130 | itb15g03720.t1 | High | 597 | 4.78 | 66.13 | Nuclear |
| ItbNAC131 | itb15g09380.t1 | High | 492 | 7.1 | 55.02 | Nuclear |
| ItbNAC132 | itb15g16380.t1 | High | 366 | 7.81 | 40.88 | Nuclear |

Table S3: The confidence level and characteristics of identified *ItfNACs*.

| Gene Name | ID | Confidence  level | ORF  (aa) | PI | Molecular weight (KD) | Subcellular location |
| --- | --- | --- | --- | --- | --- | --- |
| ItfNAC1 | itf00g01620.t1 | High | 115 | 7.07 | 12.98 | Extracellular |
| ItfNAC2 | itf00g04680.t1 | High | 143 | 9.07 | 16.58 | Extracellular |
| ItfNAC3 | itf00g18910.t1 | High | 272 | 6.75 | 31.09 | Nuclear |
| ItfNAC4 | itf00g25570.t1 | High | 320 | 4.7 | 35.56 | Nuclear |
| ItfNAC5 | itf00g28880.t1 | High | 168 | 6.27 | 19.01 | Cytoplasmic |
| ItfNAC6 | itf00g28890.t1 | High | 251 | 6.53 | 28.4 | Nuclear |
| ItfNAC7 | itf00g33690.t1 | Low | - | - | - | - |
| ItfNAC8 | itf00g38350.t1 | High | 183 | 10.08 | 21.28 | Cytoplasmic |
| ItfNAC9 | itf00g48300.t1 | High | 269 | 8.66 | 30.87 | Mitochondrial |
| ItfNAC10 | itf00g53790.t1 | Low | - | - | - | - |
| ItfNAC11 | itf00g65110.t1 | Low | - | - | - | - |
| ItfNAC12 | itf00g68040.t1 | Low | - | - | - | - |
| ItfNAC13 | itf01g00340.t1 | High | 402 | 7.65 | 44.84 | Nuclear |
| ItfNAC14 | itf01g19290.t1 | High | 342 | 8.93 | 37.75 | Nuclear |
| ItfNAC15 | itf01g25900.t1 | High | 282 | 8.66 | 31.97 | Nuclear |
| ItfNAC16 | itf01g30800.t1 | High | 463 | 4.42 | 52.78 | Nuclear |
| ItfNAC17 | itf01g30810.t1 | High | 585 | 4.44 | 65.96 | Nuclear |
| ItfNAC18 | itf01g34500.t1 | High | 438 | 4.4 | 48.27 | Nuclear |
| ItfNAC19 | itf02g00760.t1 | High | 331 | 6.91 | 37.44 | Nuclear |
| ItfNAC20 | itf02g03850.t1 | High | 332 | 8.7 | 37.26 | Nuclear |
| ItfNAC21 | itf02g06750.t1 | High | 400 | 5.06 | 44.82 | Nuclear |
| ItfNAC22 | itf02g06760.t1 | High | 535 | 4.38 | 59.83 | Cytoplasmic |
| ItfNAC23 | itf02g10140.t1 | High | 290 | 8.74 | 33.44 | Nuclear |
| ItfNAC24 | itf02g12660.t1 | High | 293 | 8.79 | 32.94 | Nuclear |
| ItfNAC25 | itf02g13580.t1 | High | 251 | 6.62 | 27.98 | Nuclear |
| ItfNAC26 | itf02g14480.t1 | High | 210 | 9.85 | 24.51 | Nuclear |
| ItfNAC27 | itf02g15080.t1 | High | 360 | 8.11 | 39.71 | Chloroplast |
| ItfNAC28 | itf02g15450.t1 | High | 337 | 8.4 | 37.6 | Nuclear |
| ItfNAC29 | itf02g15460.t1 | High | 353 | 9.1 | 38.95 | Nuclear |
| ItfNAC30 | itf03g00520.t1 | High | 390 | 4.87 | 43.11 | Nuclear |
| ItfNAC31 | itf03g01630.t1 | High | 297 | 6.04 | 33.49 | Nuclear |
| ItfNAC32 | itf03g04990.t1 | High | 353 | 5.96 | 40.2 | Nuclear |
| ItfNAC33 | itf03g06200.t1 | High | 258 | 8.93 | 29.36 | Cytoplasmic |
| ItfNAC34 | itf03g07030.t1 | High | 356 | 5.18 | 40.34 | Nuclear |
| ItfNAC35 | itf03g09920.t1 | Low | - | - | - | - |
| ItfNAC36 | itf03g18330.t1 | High | 474 | 6.96 | 52.43 | Nuclear |
| ItfNAC37 | itf03g28670.t1 | High | 302 | 8.12 | 34.79 | Nuclear |
| ItfNAC38 | itf04g18350.t1 | High | 203 | 8.59 | 23.36 | Nuclear |
| ItfNAC39 | itf04g21110.t1 | High | 153 | 9.18 | 18.01 | Nuclear |
| ItfNAC40 | itf04g26240.t1 | High | 291 | 6.43 | 33.03 | Cytoplasmic |

Table S3 continued

| Gene Name | ID | Confidence level | ORF  (aa) | PI | Molecular weight (KD) | Subcellular location |
| --- | --- | --- | --- | --- | --- | --- |
| ItfNAC41 | itf04g32060.t1 | High | 198 | 9.41 | 22.91 | Nuclear |
| ItfNAC42 | itf04g33330.t1 | High | 250 | 5.78 | 29.28 | Cytoplasmic |
| ItfNAC43 | itf04g33340.t1 | High | 327 | 5 | 37.3 | Nuclear |
| ItfNAC44 | itf04g33740.t1 | High | 521 | 6.23 | 59.04 | Cytoplasmic |
| ItfNAC45 | itf04g33750.t1 | Low | - | - | - | - |
| ItfNAC46 | itf05g00220.t1 | High | 344 | 7.82 | 39.7 | Nuclear |
| ItfNAC47 | itf05g00240.t1 | High | 381 | 7.52 | 43.33 | Nuclear |
| ItfNAC48 | itf05g00910.t1 | High | 243 | 9.37 | 27.41 | Nuclear |
| ItfNAC49 | itf05g01820.t1 | High | 342 | 6.61 | 39.23 | Nuclear |
| ItfNAC50 | itf05g04450.t1 | High | 284 | 4.2 | 32.21 | Nuclear |
| ItfNAC51 | itf05g04480.t1 | High | 283 | 4.27 | 32.16 | Nuclear |
| ItfNAC52 | itf05g09070.t1 | High | 282 | 4.36 | 31.99 | Cytoplasmic |
| ItfNAC53 | itf05g09150.t1 | High | 474 | 6.63 | 53.33 | Nuclear |
| ItfNAC54 | itf05g12720.t1 | High | 577 | 4.66 | 64.6 | Nuclear |
| ItfNAC55 | itf05g16070.t1 | High | 294 | 6.6 | 33.33 | Cytoplasmic |
| ItfNAC56 | itf05g16640.t1 | High | 458 | 4.07 | 50.19 | Nuclear |
| ItfNAC57 | itf05g16650.t1 | High | 442 | 4.2 | 48.79 | Nuclear |
| ItfNAC58 | itf05g20060.t1 | High | 580 | 4.82 | 63.92 | Nuclear |
| ItfNAC59 | itf05g21000.t1 | High | 271 | 6.63 | 30.76 | Chloroplast |
| ItfNAC60 | itf05g21160.t1 | High | 588 | 4.49 | 64.5 | Nuclear |
| ItfNAC61 | itf05g23010.t1 | High | 685 | 6.51 | 78 | Nuclear |
| ItfNAC62 | itf05g25290.t1 | High | 322 | 7.21 | 35.82 | Chloroplast |
| ItfNAC63 | itf05g27190.t1 | High | 352 | 8.66 | 40.5 | Nuclear |
| ItfNAC64 | itf06g13280.t1 | High | 359 | 7.24 | 40.74 | Nuclear |
| ItfNAC65 | itf06g14060.t1 | High | 310 | 7.23 | 35.17 | Nuclear |
| ItfNAC66 | itf06g14780.t1 | High | 294 | 6.37 | 33.42 | Nuclear |
| ItfNAC67 | itf06g15150.t1 | High | 303 | 7.17 | 35.08 | Nuclear |
| ItfNAC68 | itf06g15330.t1 | High | 356 | 8.13 | 38.83 | Nuclear |
| ItfNAC69 | itf06g19310.t1 | High | 490 | 4.4 | 53.77 | Nuclear |
| ItfNAC70 | itf06g19320.t1 | High | 328 | 7.62 | 37.61 | Nuclear |
| ItfNAC71 | itf06g19970.t1 | High | 835 | 4.59 | 93.14 | Chloroplast |
| ItfNAC72 | itf06g22360.t1 | High | 400 | 4.77 | 44.49 | Nuclear |
| ItfNAC73 | itf06g26120.t1 | High | 330 | 6.83 | 37.29 | Nuclear |
| ItfNAC74 | itf07g00540.t1 | High | 265 | 6.78 | 30.49 | Nuclear |
| ItfNAC75 | itf07g00590.t1 | High | 392 | 7.5 | 43.99 | Nuclear |
| ItfNAC76 | itf07g03260.t1 | High | 354 | 7.12 | 39.54 | Nuclear |
| ItfNAC77 | itf07g04760.t1 | High | 356 | 5 | 41.43 | Nuclear |
| ItfNAC78 | itf07g04770.t1 | High | 308 | 4.73 | 35.76 | Nuclear |
| ItfNAC79 | itf07g04780.t1 | High | 178 | 10.57 | 21.18 | Mitochondrial |
| ItfNAC80 | itf07g04790.t1 | High | 155 | 10.61 | 18.66 | Mitochondrial |

Table S3 continued

| Gene  Name | ID | Confidence  level | ORF  (aa) | PI | Molecular weight (KD) | Subcellular location |
| --- | --- | --- | --- | --- | --- | --- |
| ItfNAC81 | itf07g04800.t1 | High | 340 | 4.78 | 39.04 | Nuclear |
| ItfNAC82 | itf07g04810.t1 | High | 411 | 4.99 | 47.18 | Nuclear |
| ItfNAC83 | itf07g04820.t1 | High | 334 | 4.56 | 38.26 | Nuclear |
| ItfNAC84 | itf07g04830.t1 | High | 339 | 5.55 | 39.59 | Nuclear |
| ItfNAC85 | itf07g04840.t1 | High | 307 | 4.97 | 35.65 | Nuclear |
| ItfNAC86 | itf07g04850.t1 | High | 337 | 4.57 | 38.94 | Nuclear |
| ItfNAC87 | itf07g11480.t1 | High | 332 | 9.21 | 37.96 | Nuclear |
| ItfNAC88 | itf07g17390.t1 | High | 278 | 6.43 | 31.65 | Cytoplasmic |
| ItfNAC89 | itf07g17700.t1 | High | 288 | 4.59 | 32.97 | Nuclear |
| ItfNAC90 | itf07g21970.t1 | High | 296 | 7.98 | 33.58 | Nuclear |
| ItfNAC91 | itf07g22170.t1 | High | 390 | 7.46 | 43.05 | Nuclear |
| ItfNAC92 | itf08g01730.t1 | High | 349 | 5.43 | 39.09 | Nuclear |
| ItfNAC93 | itf08g04440.t1 | High | 278 | 6.05 | 31 | Chloroplast |
| ItfNAC94 | itf08g07530.t1 | High | 201 | 9.97 | 23.37 | Extracellular |
| ItfNAC95 | itf08g07600.t1 | High | 314 | 6.83 | 35.49 | Nuclear |
| ItfNAC96 | itf08g10040.t1 | High | 206 | 8.73 | 23.03 | Extracellular |
| ItfNAC97 | itf08g10050.t1 | High | 210 | 8.71 | 23.56 | Chloroplast |
| ItfNAC98 | itf08g10060.t1 | High | 215 | 9.19 | 23.68 | Chloroplast |
| ItfNAC99 | itf08g10090.t1 | High | 196 | 7.67 | 22.03 | Extracellular |
| ItfNAC100 | itf08g10100.t1 | High | 138 | 9.12 | 15.61 | Extracellular |
| ItfNAC101 | itf08g12580.t1 | High | 90 | 4.39 | 10.13 | Cytoplasmic |
| ItfNAC102 | itf09g00190.t1 | High | 345 | 6.65 | 38.76 | Nuclear |
| ItfNAC103 | itf09g03660.t1 | High | 197 | 4.8 | 22.28 | Nuclear |
| ItfNAC104 | itf09g06980.t1 | High | 300 | 8.24 | 33.52 | Nuclear |
| ItfNAC105 | itf09g07000.t1 | High | 271 | 8.56 | 30.73 | Nuclear |
| ItfNAC106 | itf09g10480.t1 | High | 325 | 6.52 | 36.17 | Chloroplast |
| ItfNAC107 | itf09g13340.t1 | High | 261 | 7.38 | 29.32 | Nuclear |
| ItfNAC108 | itf09g25220.t1 | High | 342 | 6.78 | 38.8 | Nuclear |
| ItfNAC109 | itf09g26910.t1 | High | 400 | 6.95 | 45.24 | Nuclear |
| ItfNAC110 | itf10g00780.t1 | High | 343 | 8.64 | 37.61 | Nuclear |
| ItfNAC111 | itf10g07790.t1 | High | 469 | 5.37 | 53.51 | Nuclear |
| ItfNAC112 | itf10g14260.t1 | High | 330 | 5.66 | 38.44 | Nuclear |
| ItfNAC113 | itf10g14360.t1 | High | 487 | 4.17 | 53.24 | Nuclear |
| ItfNAC114 | itf10g16540.t1 | High | 380 | 6.66 | 42.54 | Nuclear |
| ItfNAC115 | itf10g16720.t1 | High | 622 | 6.08 | 68.42 | Nuclear |
| ItfNAC116 | itf10g20230.t1 | High | 328 | 8.85 | 36.22 | Nuclear |
| ItfNAC117 | itf10g22560.t1 | High | 197 | 4.83 | 22.6 | Nuclear |
| ItfNAC118 | itf11g03550.t1 | High | 189 | 8.63 | 20.97 | Nuclear |
| ItfNAC119 | itf11g03560.t1 | High | 251 | 6.77 | 28.4 | Cytoplasmic |
| ItfNAC120 | itf11g07600.t1 | High | 399 | 5.25 | 44.5 | Nuclear |

Table S3 continued

| Gene  Name | ID | Confidence  level | ORF  (aa) | PI | Molecular weight (KD) | Subcellular location |
| --- | --- | --- | --- | --- | --- | --- |
| ItfNAC121 | itf11g11490.t1 | High | 246 | 4.59 | 28.54 | Nuclear |
| ItfNAC122 | itf11g18490.t1 | High | 281 | 7.18 | 31.58 | Nuclear |
| ItfNAC123 | itf12g00300.t1 | High | 486 | 4.61 | 55.95 | Nuclear |
| ItfNAC124 | itf12g02830.t1 | High | 369 | 5.98 | 42.34 | Nuclear |
| ItfNAC125 | itf12g06530.t1 | High | 286 | 6.51 | 32.12 | Nuclear |
| ItfNAC126 | itf12g06670.t1 | High | 357 | 7.63 | 40.01 | Nuclear |
| ItfNAC127 | itf12g08650.t1 | High | 297 | 5.77 | 34.67 | Cytoplasmic |
| ItfNAC128 | itf12g08670.t1 | High | 205 | 5.08 | 24.62 | Cytoplasmic |
| ItfNAC129 | itf12g08680.t1 | High | 179 | 8.55 | 21 | Cytoplasmic |
| ItfNAC130 | itf12g12810.t1 | High | 374 | 4.89 | 42.17 | Nuclear |
| ItfNAC131 | itf12g15230.t1 | High | 175 | 10.23 | 20.63 | Nuclear |
| ItfNAC132 | itf12g24590.t1 | High | 354 | 6.83 | 40.56 | Nuclear |
| ItfNAC133 | itf12g26670.t1 | High | 627 | 4.78 | 68.81 | Nuclear |
| ItfNAC134 | itf12g27410.t1 | High | 300 | 7.16 | 34.7 | Cytoplasmic |
| ItfNAC135 | itf12g27600.t1 | High | 446 | 4.48 | 48.81 | Nuclear |
| ItfNAC136 | itf13g04700.t1 | High | 345 | 6.92 | 40.01 | Nuclear |
| ItfNAC137 | itf13g13630.t1 | High | 101 | 9.99 | 11.52 | Nuclear |
| ItfNAC138 | itf13g13960.t1 | Low | - | - | - | - |
| ItfNAC139 | itf13g16970.t1 | High | 256 | 8.84 | 28.71 | Nuclear |
| ItfNAC140 | itf13g22540.t1 | High | 474 | 4.85 | 53.88 | Nuclear |
| ItfNAC141 | itf14g00550.t1 | High | 243 | 5.74 | 27.62 | Cytoplasmic |
| ItfNAC142 | itf14g04780.t1 | High | 298 | 5.14 | 33.34 | Nuclear |
| ItfNAC143 | itf14g04910.t1 | High | 348 | 4.68 | 38.92 | Nuclear |
| ItfNAC144 | itf14g04920.t1 | High | 327 | 4.79 | 36.96 | Nuclear |
| ItfNAC145 | itf14g04960.t1 | High | 349 | 4.6 | 39.08 | Nuclear |
| ItfNAC146 | itf14g12330.t1 | High | 486 | 5.09 | 54.26 | Nuclear |
| ItfNAC147 | itf14g17960.t1 | High | 258 | 6.62 | 29.06 | Cytoplasmic |
| ItfNAC148 | itf15g01560.t1 | High | 260 | 5.45 | 29.44 | Nuclear |
| ItfNAC149 | itf15g03430.t1 | High | 626 | 5.27 | 69.47 | Nuclear |
| ItfNAC150 | itf15g08990.t1 | High | 488 | 7.3 | 54.43 | Nuclear |
| ItfNAC151 | itf15g15530.t1 | High | 366 | 8.22 | 40.8 | Nuclear |
